# Protective Effect of Ocotillol, the Derivate of Ocotillol-Type Saponins in *Panax* Genus, against Acetic Acid-Induced Gastric Ulcer in Rats Based on Untargeted Metabolomics

**DOI:** 10.3390/ijms21072577

**Published:** 2020-04-08

**Authors:** Cuizhu Wang, Yuze Yuan, He Pan, Alan Chen-Yu Hsu, Jinluan Chen, Jinping Liu, Pingya Li, Fang Wang

**Affiliations:** 1 Department of Pathogen Biology, College of Basic Medical Sciences, Jilin University, Changchun 130021, China; wangcuizhu@jlu.edu.cn (C.W.); yuanyz19@mails.jlu.edu.cn (Y.Y.); panhe18@mails.jlu.edu.cn (H.P.); 2 School of Pharmaceutical Sciences, Jilin University, Fujin Road 1266, Changchun 130021, China; liujp@jlu.edu.cn (J.L.); lipy@jlu.edu.cn (P.L.); 3 Priority Research Centre for Healthy Lungs, Faculty of Health and Medicine, The University of Newcastle, Newcastle, NSW 2305, Australia; alan.hsu@newcastle.edu.au; 4 Department of Internal Medicine, Erasmus MC, University Medical Center Rotterdam, 3031RM Rotterdam, The Netherlands; j.chen@erasmusmc.nl

**Keywords:** Ocotillol, gastric ulcer, metabolomics, UPLC-QTOF-MS

## Abstract

Gastric ulcer (GU), a prevalent digestive disease, has a high incidence and is seriously harmful to human health. Finding a natural drug with a gastroprotective effect is needed. Ocotillol, the derivate of ocotillol-type saponins in the *Panax* genus, possesses good anti-inflammatory activity. The study aimed to investigate the gastroprotective effect of ocotillol on acetic acid-induced GU rats. The serum levels of endothelin-1 (ET-1) and nitric oxide (NO), the gastric mucosa levels of epidermal growth factor, superoxide dismutase and NO were assessed. Hematoxylin and eosin staining of gastric mucosa for pathological changes and immunohistochemical staining of ET-1, epidermal growth factor receptors and inducible nitric oxide synthase were evaluated. A UPLC-QTOF-MS-based serum metabolomics approach was applied to explore the latent mechanism. A total of 21 potential metabolites involved in 7 metabolic pathways were identified. The study helps us to understand the pathogenesis of GU and to provide a potential natural anti-ulcer agent.

## 1. Introduction

Gastric ulcer (GU), characterized by rhythmic burning pain in upper abdomen, often occurs on the surface of gastric mucosa with high incidence and could cause bleeding, stenosis, perforation and pyloric obstruction. It is a kind of precancerous gastric cancer disease and plays a vital role in the occurrence and process of intestinal-type gastric cancer [1,2]. The main reasons for GU formation include infection of Helicobacter pylori bacteria, gastric hyperacidity, local ischemia or damaged barrier effect of the gastric mucosa [3]. The ultimate formation of a gastric ulcer is due to the digestion of gastric acid and pepsin, and gastric acid is the decisive factor for the occurrence of the ulcer. Acetic acid-induced ulcers, one of the standard animal models, is widely used to conduct pharmacological and pathophysiological studies on gastric ulcers [4,5,6,7]. Common evaluation indexes for GU include inflammatory cytokines, reactive oxygen radicals, and the local blood supply of the gastric tissue [8]. At present, proton pump inhibitors, H_2_-receptor antagonists and *Helicobacter pylori* eradication therapy are widely used in GU treatment [9]. However, in spite of their satisfactory therapeutic effects, there were some associated undesirable adverse drug reactions, drug resistance and high recurrence rates after treatment. Thus, a more ideal antiulcer drug is urgently needed. The discovery of natural products may afford a safer and more effective alternative with fewer side effects.

**Figure 1:**
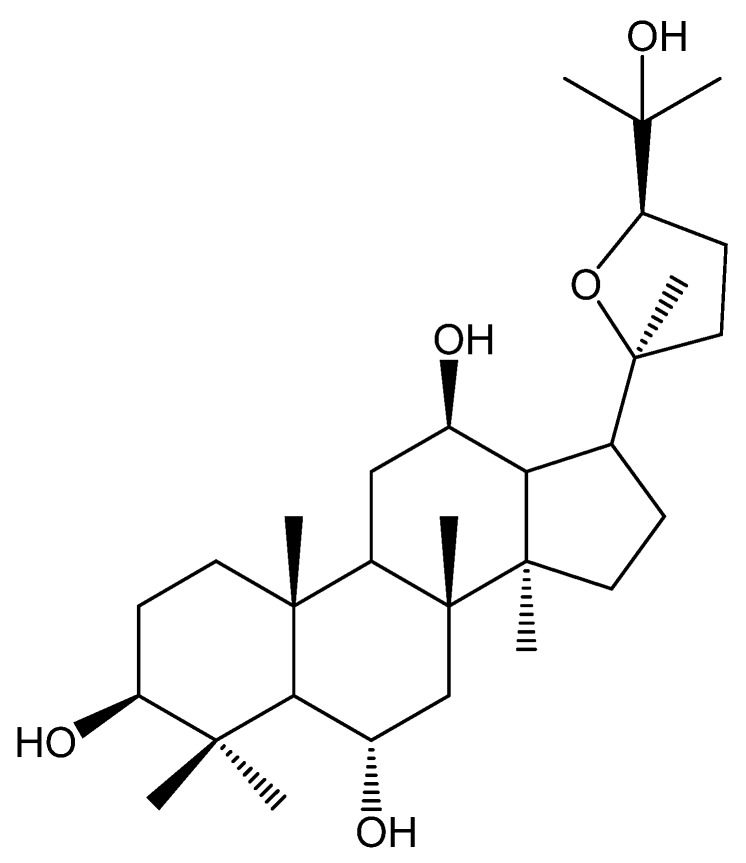
The structure of ocotillol.

In genus *Panax*, *Panax. quinquefolium* L., *Panax vietnamensis* Ha et Grushv. and *Panax japonicus* C. A. Mey. have been widely used as both medicinal and dietary supplements, which were rich in ocotillol-type saponins and have multiple biological activities such as a protective effect against gastric lesions [10], anti-inflammatory and anti-oxidation effects [11,12,13]. All of the saponins, such as pseudoginsenoside F11, -RT5, -RT4 and majonoside-R2, have the common sapogenin (namely ocotillol, Figure 1). Ocotillol is also the major metabolite of ocotillol-type saponins after oral administration [14]. It was also reported to exert an anti-inflammatory effect and ameliorate 2,4,6-trinitro-benzenesulfonic acid-induced colitis [15,16,17]. While there was no report on the gastroprotective effect of ocotillol.

Metabolomics, a systematic study of the metabolites in biological samples, was prospective for discovering the pathways linked to disease processes and elucidating the mechanism of drugs [18,19,20]. Based on ultra-high-performance liquid chromatography combined with quadrupole time-of-flight mass spectrometry (UPLC-QTOF-MS), multivariate statistical analysis, such as principle component analysis (PCA) and orthogonal projections to latent structures discriminant analysis (OPLS-DA), was widely applied in metabolomics analysis to screen and identification of the functional metabolites.

In the present study, the metabolomics strategy based on UPLC-QTOF-MS was used to investigate the anti-GU effect of ocotillol in an acetic-acid-induced rat model, and to illustrate the potential biomarkers and related metabolic pathways. The results could help us to understand the pathogenesis of GU and to provide a potential natural anti-ulcer agent.

## 2. Results

### 2.1. Gastroprotective Effect

#### 2.1.1. Body Weights of the Rats

**Figure 2:**
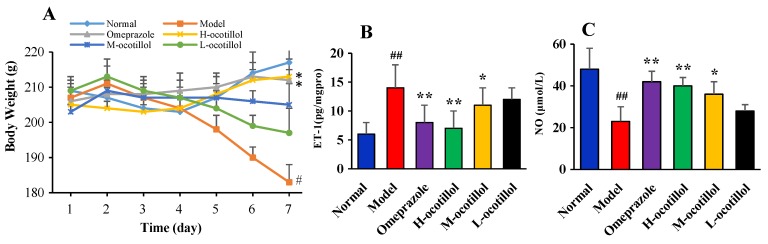
(**A**) Body weights of animals, (**B**) ET-1 levels and (**C**) NO levels in serum. (Compared with normal group, # *p* < 0.05, ## *p* < 0.01; compared with model group, * *p* < 0.05, ** *p* < 0.01).

As shown in Figure 2A, during the 7 days, the body weights of the rats in the model group severely decreased, and the body weights of the rats in the L-ocotillol group also reduced. While the M-ocotillol group showed a slight increase, both omeprazole and H-ocotillol groups showed a gradual increase. On the 7th day, the significant decreased weights of the model group were compared with the normal group (*p* < 0.05), and there was a significant weight rise after omeprazole or H-ocotillol treatment compared with the model group (*p* < 0.05).

#### 2.1.2. Endothelin-1 (ET-1) and Nitric Oxide (NO) Levels in Serum

As shown in Figure 2B,C, acetic acid could remarkably change serum ET-1 and NO levels in the model group compared with the normal group (*p* < 0.01), while ocotillol could re-regulate the ET-1 and NO levels in a dose-dependent manner compared with the model group (*p* < 0.05, *p* < 0.01), and the H-ocotillol showed a similar effect to that of omeprazole.

#### 2.1.3. Epidermal Growth Factor(EGF), Superoxide Dismutase (SOD), and NO Levels in Gastric Mucosa

**Figure 3:**
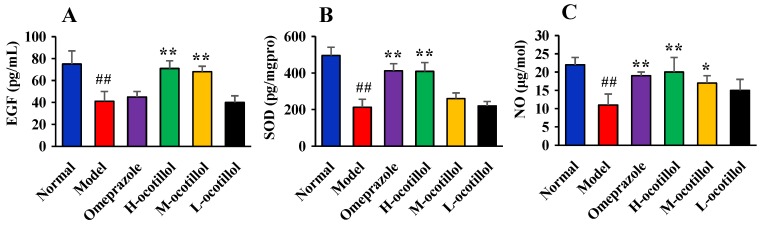
(**A**) epidermal growth factor (EGF), (**B**) SOD and (**C**) NO levels in gastric mucosa. (Compared with the normal group, ## *p* < 0.01; compared with the model group, * *p* < 0.05, ** *p* < 0.01).

As shown in Figure 3, acetic acid could remarkably decrease mucosa EGF, SOD, and NO levels in the model group compared with the normal group (*p* < 0.01), while H-ocotillol could increase mucosa EGF, SOD, and NO levels compared with the model group (*p* < 0.01). In addition, M-ocotillol could also significantly increase the EGF level (*p* < 0.01), and increase the NO level (*p* < 0.05). For the SOD and NO levels, the H-ocotillol showed a similar effect to that of omeprazole, but for the EGF levels, omeprazole showed little effect.

#### 2.1.4. Histological and Immunohistochemical Analysis

**Figure 4:**
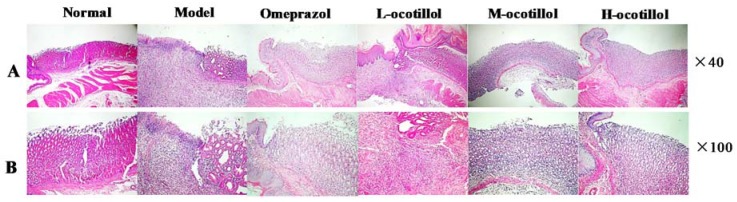
Hematoxylin and eosin (H&E) staining of the gastric mucosa. (**A**×40; **B**×100).

**Figure 5:**
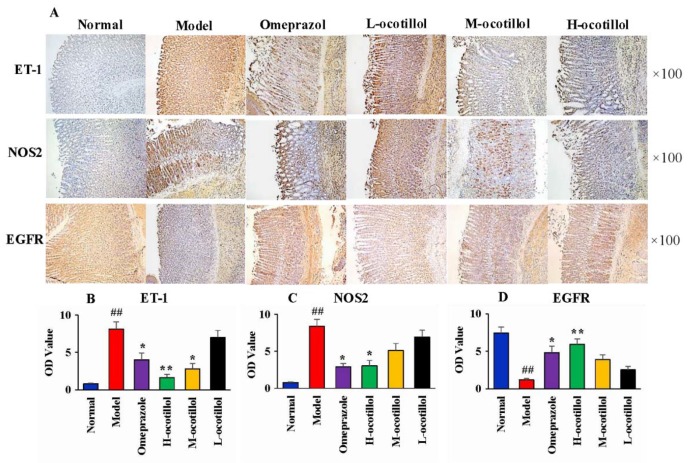
(**A**) Immunohistochemical analysis of ET-1, NOS2 and EGFR in different groups (×100). OD values of (**B**) ET-1, (**C**) NOS2 and (**D**) EGFR (Compared with normal group, ^##^ *p* < 0.01; compared with model group, * *p* < 0.05, ** *p* < 0.01). (OD value: optical density value)

Histopathological results showed that the normal stomach tissue included the serosa, mucosa, muscularis and submucosa, the gland was closely arranged, the epitheliums maintained their integrity, and there was no congestion and edema. In the stomach tissue in the GU model group, severe histopathological changes, including several large ulcers, mucosa lesions, atrophic and disorganized gland, infiltrated inflammatory cells, mucosa congestion and edema, were clearly observed. In the H, M-ocotillol groups and omeprazole group, the GU-related histopathologies were alleviated, and the histopathological results were similar to those of the normal group. The GU-related histopathology in the L-ocotillol group showed no evident improvement (Figure 4). In the immunohistochemical analysis, compared with the normal group, the levels of ET-1 and inducible nitric oxide synthase (NOS2) in model group significantly increased (*p* < 0.01), and the level of epidermal growth factor receptor (EGFR) in the model group significantly decreased (*p* < 0.01). On one hand, ocotillol could regulate the levels of ET-1, NOS2 and EGFR in a dose-dependent manner, namely H, M-ocotillol demonstrated a better effect than the L-ocotillol. On the other hand, H-ocotillol demonstrated a better effect on both ET-1 and EGFR than the positive drug (omeprazole) (Figure 5).

#### 2.1.5. Molecular Docking

**Figure 6:**
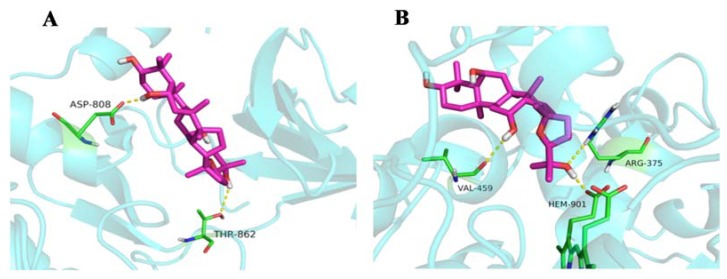
Docking of ocotillol in (**A**) 3RCD and (**B**) 3EAI.

The action mode, such as localized binding sites in the active pocket or ocotillol’s structural configuration, of ocotillol on EGF and NOS2 is shown in Figure 6. Two hydrogen bonds were observed with ocotillol at ASP-808, THR-862 residues in EGF. Three hydrogen bonds were observed at VAL-459, HEM-901 and ARG-375 residues in NOS2.

### 2.2. Metabolomics Study

#### 2.2.1. Validation of UPLC-QTOF-MS

**Figure 7:**
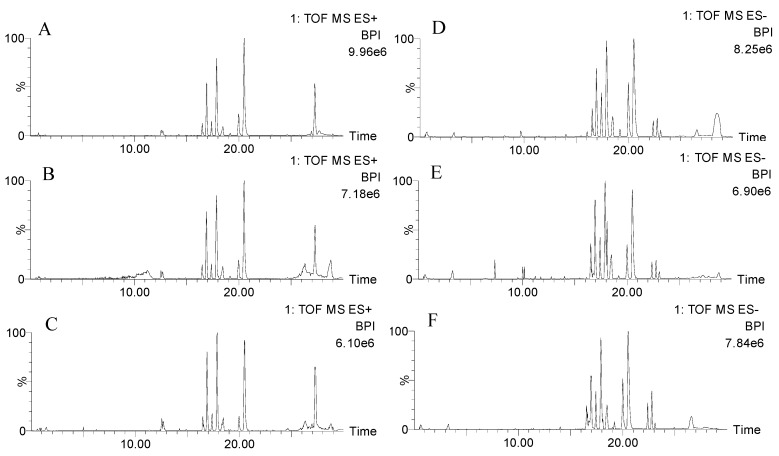
The representative base peak intensity (BPI) chromatograms of serum samples of normal (**A**), model (**B**) and H-ocotillol (**C**) groups in positive modes; and those of normal (**D**), model (**E**) and H-ocotillol (**F**) groups in negative modes.

UPLC-QTOF-MS was used to obtain the metabolic characteristics in positive and negative modes of the normal group, model group and H-ocotillol groups. Here, the representative base peak intensity (BPI) chromatograms of each group of serum samples are shown in Figure 7. In order to monitor the consistency of the system, the quality control (QC) sample was run randomly covering the whole analysis process, eight ions were monitored as the extracted ion chromatographic peaks, which were selected from different spectral regions. The exact mass/retention time pairs of these ions in serum were as follows: *m/z* 203.0530, 0.55 min; *m/z* 582.2961, 5.04 min; *m/z* 790.1797, 11.30 min; *m/z* 274.2748, 12.55 min; *m/z* 341.1584, 13.50 min; *m/z* 520.3380, 16.92 min; *m/z* 524.3699, 20.51 min; *m/z* 338.3431, 27.30 min in ESI+ mode; and *m/z* 197.8078, 0.52 min; *m/z* 322.9478, 3.27 min; *m/z* 630.7985, 5.04 min; *m/z* 991.5497, 7.34 min; *m/z* 564.3334, 16.52 min; *m/z* 540.3310, 17.90 min; *m/z* 568.3635, 20.51 min; *m/z* 279.2317, 23.12 min in ESI− mode. The relative standard deviations (RSDs) of the retention times and the peak intensities of the eight ions were 0.56%–3.14% and 0.67%–7.56%, respectively.

The injection precision was assessed by detecting five consecutive injections of the QC sample. For the selected eight ions, the RSDs of peak intensity ranged from 0.82% to 2.79% and RSDs of retention time ranged from 0.04% to 0.31% in ESI+. In ESI−, the RSDs of peak intensity and retention time were from 0.21% to 3.12% and from 0.09% to 0.54%, respectively.

The reproducibility of sample preparation was evaluated by analyzing five parallel replicates of a serum sample. The RSDs of the retention time of the selected eight ions were 0.07%–1.34%, those of peak intensities were 1.41%–3.99% in ESI+, while they were 0.11%–0.52% and 0.18%–3.01% in ESI−.

The post-preparation stability of the sample was estimated by detecting one sample that was placed in the autosampler held at 16 °C for 0, 4, 8, 10, and 12 h [21]. For the selected eight ions in ESI+ and ESI− modes, the RSDs of the retention time were 0.11%~0.61% and 0.23%–0.74%, and the RSDs of peak intensity were 1.98%–4.87% and 0.72%–5.12%, respectively.

The above validation results showed that the UPLC-QTOF-MS method exerted good precision, reproducibility and stability.

#### 2.2.2. Identification of the Differential Metabolites and Metabolic Pathways

**Figure 8:**
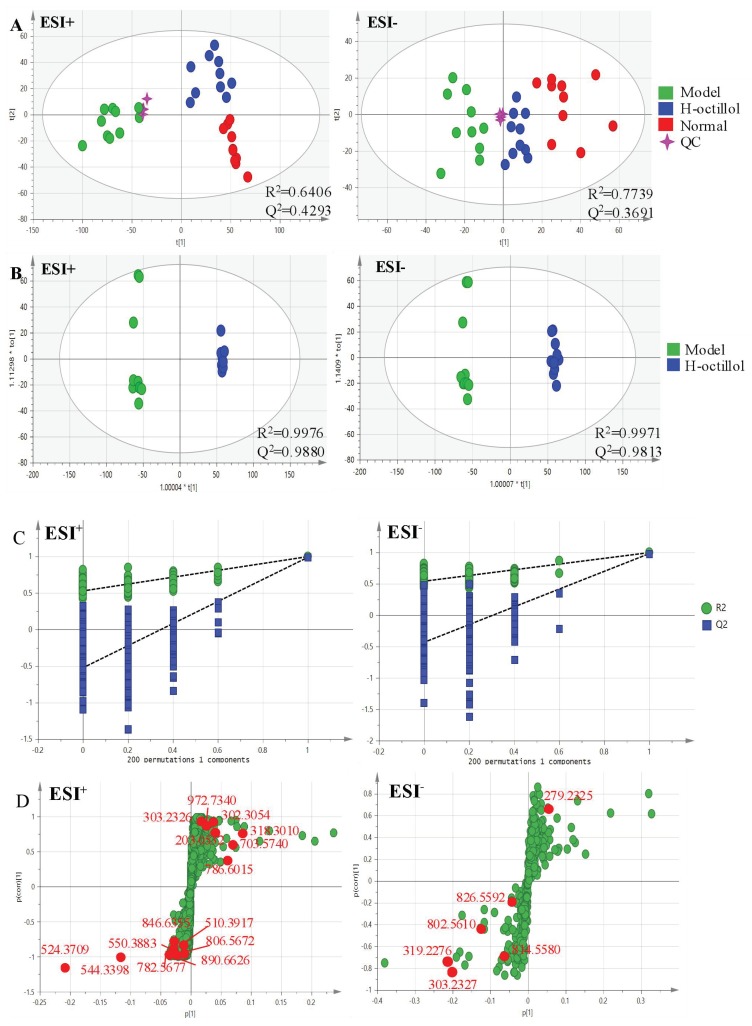
(**A**) PCA score plots of serum metabolic profiling of normal, model and H-ocotillol groups; (**B**) OPLS-DA score plots of serum metabolic profiling of model and H-ocotillol groups; (**C**) The permutations plots of the OPLS-DA models; (**D**) OPLS-DA S-plots of serum metabolic profiling.

**Figure 9:**
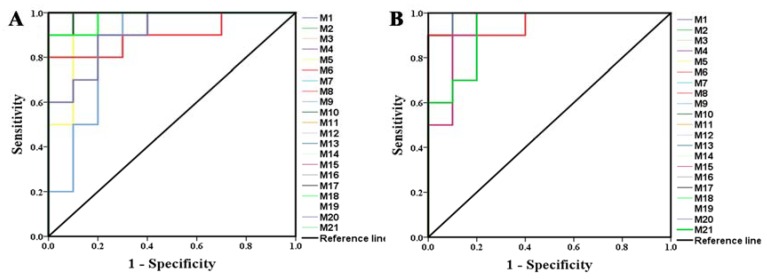
The predictive receiver operating characteristic (ROC) curves generated using 21 biomarkers contributing to (**A**) gastric ulcer progress between the model group and the normal group, (**B**) ocotillol treatment between the model group and H-ocotillol group (the numbers are consistent with No. in Table 1).

**Figure 10:**
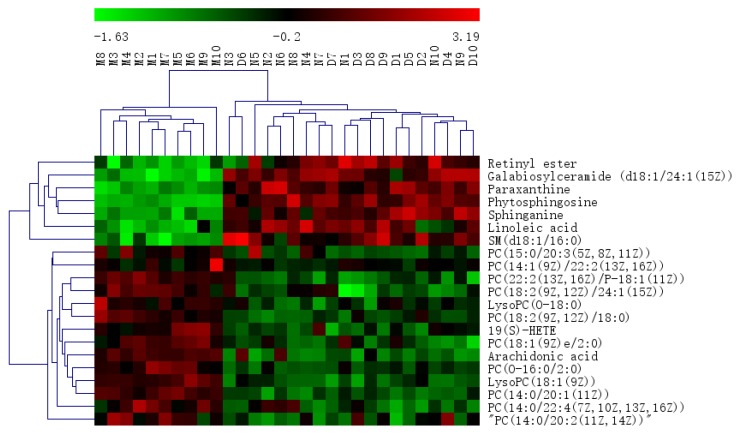
The heatmap of all potential biomarkers.

**Figure 11:**
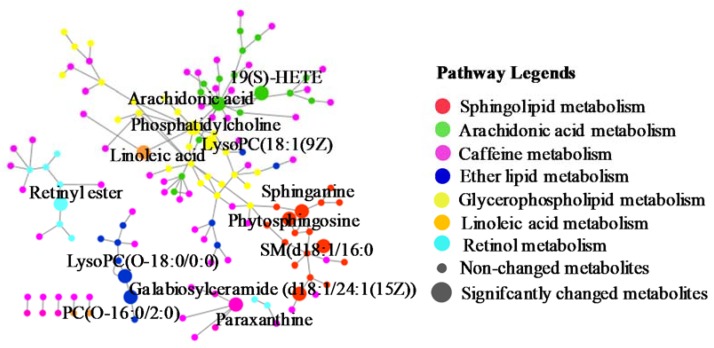
The metabolic pathways.

Pareto scaling, one of the major approaches to multiobjective programming, was applied to establish the PCA, OPLS-DA and S-plot in the study. PCA score 2D plots were established in both ESI+ and ESI- modes (Figure 8A). Each spot of PCA score plots represented a sample. QC samples were tightly clustered and located in the middle of three groups, which indicated that the stability of the system was satisfactory. The samples from different groups were generally clustered together, which showed that similarity existed in each group. Furthermore, a clear separation of three groups was observed, indicating that these three groups were differential. Additionally, the H-ocotillol group was located between the model and normal group, which indicated that a high dose of ocotillol may improve the metabolic disturbances in GU model rats. Aiming to further find potential biomarkers that made remarkable contribution to the metabolic distinction, OPLS-DA models were established in both ESI+ and ESI- modes (Figure 8B). Each sample was represented as one spot in score plots. The satisfactory parameters (R^2^ and Q^2^) indicated the model had good prediction ability and reliability in both ESI+ and ESI- modes. The models were valid since all Q^2^-values to the left of the permutation plots were lower than the original points to the right (Figure 8C). Then, the S-plots were generated to identify the differential metabolites (Figure 8D). Each spot in the S-plots represented a variable. The further away they were from the origin, the more significantly the spots contributed to the clustering of the model group and H-ocotillol group. A total of 21 robust endogenous metabolites were identified as candidate biomarkers (marked in red in S-plots) in serum samples (Table 1). The MS/MS spectra of potential markers, standards and the results of the related database of HMDB or METLIN were shown in Supplementary Materials (Figures S1–S21). The predictive ROC curves were generated using the 21 candidate biomarkers. The ROC curves between the model and normal groups showed that all of them were potential diagnostic markers for GU (Figure 9A, Table 2). The other ROC curves were generated between model and H-ocotillol groups, indicating that all of the 21 metabolites contributed to ocotillol treatment (Figure 9B, Table 2). The heatmap was generated to visualize and characterize the relative abundance of the biomarkers in different groups; green represented low abundance and red represented high abundance (Figure 10). The metabolic network of the biomarkers was established (Figure 11), which clearly showed that H-ocotillol could regulate the alterations in caffeine metabolism (CM), sphingolipid metabolism (SphM), arachidonic acid metabolism (AM), linoleic acid metabolism (LM), glycerophospholipid metabolism (GlyM), retinol metabolism (RM), and ether lipid metabolism (EM) (Table 3).

## 3. Discussion

Due to various exogenous damaging factors including smoking, stress, poor diet, excessive drinking and prolonged ingestion of nonsteroidal antiinflammation drugs, the incidence of GU increases year by year. A novel antiulcer drug is needed. Ocotillol, the saponin-derived sapogenin in *Panax* genus, with the good anti-inflammatory activity, could be either isolated from *Panax* or prepared with ocotillol-type saponins. Majonoside R2 and pseudoginsenosides F11, RT5, RT4 are all ocotillol-type ginsenosides. Furthermore, ocotillol is one of the major metabolites of these ocotillol-type saponins after oral administration. For example, majonoside R2 was metabolized to ocotillol via pseudoginsenoside RT4, and these ginsenosides all demonstrated inhibitory effects against Th17 cell differentiation. However, ocotillol showed the highest inhibitory effect among these three ginsenosides [15]. Another example, pseudoginsenosides F11, was metabolized to ocotillol via pseudoginsenoside RT5 [32]. Furthermore, ocotillol could enhance neuronal activity [33], which was similar to the effect of pseudoginsenoside F11 [34]. Based on the above reports, the ocotillol-type ginsenosides might also have a protective effect against gastric ulcers, although only ocotillol was tested in this study. However, the protective effect might be weaker than ocotillol. In the present study, the gastroprotective effect of ocotillol in acetic acid-induced GU model rats was investigated.

ET-1, an important pro-inflammatory cytokine for the contraction of blood vessels, plays a vital role in GU formation. The increasing secretion of ET-1 results in the reduced blood supply of gastric tissue and the occurrence of hypoxia, acidosis and ulcers [35]. A reduced ET-1 level is commonly associated with an increased NO level, because NO is a type of endogenous vasodilator that could inhibit the secretion of ET-1 and regulate the secretion of gastric acid. NOS2, the inducible NO synthase, is a kind of precursor of NO. The production of NOS2/NO contributes to chronic inflammation of ulcers through stimulating the synthesis of prostaglandin E2 and cyclooxygenase-2 [36]. Except for the inflammatory factors, the expression of oxygen-free radicals such as SOD was another important factor for the occurrence of injury and ulcer [37]. Furthermore, EGF is the ligand of EGFR, which is secreted in the gastrointestinal tract and could facilitate epithelial cell repair, reduce gastric acid secretion and promote the healing of ulcers [38]. Therefore, levels of ET-1, NO, NOS2, SOD, EGF and EGFR were important to assess the effect on GU. In our study, there were significant expression alterations of the above factors in the model group, while the intervention of ocotillol treatment could re-regulate these factors tending to normal levels. On one hand, H-ocotillol could increase the expression of NO, SOD, EGF and EGFR. On the other hand, H-ocotillol could decrease the expression of ET-1 and NOS2 significantly. H-ocotillol showed a similar effect to omeprazole. Molecular docking results further showed the action mode between ocotillol and NOS2 and EGF.

The metabolomic study showed that there were 21 potential biomarkers involving seven metabolic pathways.

*Arachidonic acid metabolism:* It plays a significant role in the process of inflammatory responses and is associated with gastric ulcers [39]. Arachidonic acid can be hydrolyzed, released and generated into a variety of active substances such as 19(S)-HETE [40]. In this experiment, elevated levels of 19(S)-HETE, arachidonic acid and PCs were observed in the model group, indicating the imbalance of arachinodic acid metabolism. While the increased levels could be regulated by ocotillol.

*Lipid metabolism:* PCs are also important metabolites in lipid metabolism associated with the mechanism of ulcer occurrence [41]. In the present study, four kinds of lipid metabolism including glycerophospholipid, linoleic acid, sphingolipid and ether lipid metabolisms were found. i) Glycerophospholipid metabolism: The increased LysoPC (18:1(9Z)), which could induce gastric injury and ulceration by causing impairment of the gastric mucosal barrier [42], along with the increased PCs, were observed in the model group. ii) Linoleic acid metabolism: the model group had a reduced level of linoleic acid and PCs, which indicated that the gastric ulcer could perturb linoleic acid metabolism. This is in accordance with previous studies, high levels of linoleic acid may inhibit the gastric mucosa against injury [43,44]. iii) Sphingolipid metabolism: elevated levels of PCs and decreased galabiosylceramide (d18:1/24:1(15Z)), phytosphingosine, sphinganine and SM(d18:1/16:0) were observed in the model group. Phytosphingosine was reported to exert gastro-protective activity in GU rats [45]. Sphinganine plays a significant role in regulation of cell growth, adhesion, migration, death and inflammation. SM(d18:1/16:0) could be hydrolyzed to ceramide [46], which significantly contributes to tissue damage and ulcer formation [47]. iv) Ether lipid metabolism: increased levels of PC(O-16:0/2:0) and LysoPC(O-18:0/0:0) were observed in the model group. PC(O-16:0/2:0) is a potent phospholipid activator and mediator of inflammation. LysoPC(O-18:0/0:0) is an intermediate in the pathway. In the study, ocotillol could re-regulate the expression of the above metabolites, suggesting the gastroprotective effects on GU rats.

*Caffeine metabolism:* Although we have not find the direct proof to illustrate the relationship between paraxanthine and GU, in our present study, serum levels of paraxanthine were remarkably decreased in the GU model group, but the ocotlillol treatment could increase the levels of paraxanthine compared with model group. It could be concluded that GU could cause caffeine metabolism to be perturbed.

*Retinol metabolism:* Retinyl ester is the storage form of retinol [48], which could reduce the lesion area of pressure injuries and withstand ulcer formation [49,50]. Our results revealed that the serum level of retinyl ester was markedly decreased in the model group; it was up-regulated following ocotillol treatment.

## 4. Materials and Methods

### 4.1. Materials

Ocotillol (CAS Registry Number is 5986-39-0) with a purity of 98.0%, was provided by the National and Local United Engineering R&D Center of Ginseng Innovative Drugs (Jilin, China). Omeprazole was purchased from Sigma (St. Louis, MO, USA) and used as the positive drug. ET-1, EGF, NO and SOD enzymeelinked immunosorbent assay (ELISA) kits were purchased from Longton Co. Ltd. (Shanghai, China). The antibodies of ET-1, EGFR and NOS2 were purchased from Abcam (Cambridge, MA, USA) and used for immunohistochemical staining. Methanol and acetonitrile were of UPLC-MS grade and bought from Fisher Chemical Company (Geel, Belgium). Formic acid was of UPLC-MS grade and bought from Sigma-Aldrich (St. Louis, MO, USA). Deionized water was purchased from the A.S. Watson Group Ltd (Hong Kong, China). Chloral hydrate was purchased from Biosharp Co., Ltd (Shenyang, China). Acetic acid of analytical grade (36%–38%) was purchased from J&K Technology Co., LTD. Paraxanthine (P192503, purity≥97%) was purchased from Beijing Laiyao Biological Technology Co., Ltd. (Beijing, China). Linoleic acid (L1376, purity ≥ 99%), arachidonic acid (23401, purity ≥ 97%) were purchased from Sigma-Aldrich Co., Ltd. (St. Louis, MO, USA). Phytosphingosine (101302, purity≥98%) and sphinganine (111853, purity ≥ 98%) were bought from Beijing Century Aoke Biological Technology Co., Ltd. (Beijing, China). 19(S)-HETE (111802, purity ≥ 98%) was obtained from Xi’an Ruixi Biological Technology Co.,Ltd. (Xi’an, China).

### 4.2. UPLC-QTOF-MS Conditions

A Waters Xevo G2-XS QTOF mass spectrometer (Waters Co., Milford, MA, USA.) combined with a UPLC system through an electrospray ionization (ESI) interface was used for the UPLC-QTOF-MS analysis. An ACQUITY UPLC BEH C18 (100 mm × 2.1 mm, 1.7 μm) from Waters Corporation (Massachusetts, United States) was applied for chromatographic separation. The mobile phase consists of eluent A (0.1% formic acid in water) and eluent B (0.1% formic acid in acetonitrile). The elution conditions applied were: 0~2min, 10% B; 2~26min, 10%→90% B; 26~28min, 90% B; 28~28.1min, 90%→10% B; 28.1~30min, 10% B with the flow rate at 0.4 mL/min. The column temperature was set as 31 °C and the temperature of the sample manager was set at 16 °C. Then, 10% and 90% acetonitrile aqueous solutions were used as weak and strong wash solvents respectively. The mass spectrum was obtained from 50 to 1200 Da in MS^E^ centroid mode. The optimized MS parameters were shown as follows: desolvation temperature (400 °C), source temperature (150 °C), cone voltage (40 V), capillary voltage at 2.2 kV (ESI-) and 2.6 kV (ESI+), cone gas flow (50 L/h) and desolvation gas flow (800 L/h). MSE mode was chosen with a low energy of 6 V and high energy of 20~40 V. Sodium formate was used to calibrate the mass spectrometer in the range of 50 to 1200 Da in order to ensure the mass reproducibility and accuracy. Leucine enkephalin (m/z 556.2771 in ESI+ and 554.2615 in ESI-) was applied as external reference for Lock SprayTM injected at a flow of 10 µL/min. The QC sample was injected randomly 4 times throughout the whole worklist. All of the volume injections of the samples and QC were 5 µL per run. Data recording was performed on a MassLynx V4.1 workstation (Waters, Manchester, UK).

### 4.3. Experimental Design

Animal experiments were performed according to the protocols approved by the Review Committee of Animal Care and Use of Jilin University. Male Wistar rats, weighing 180~220 g, were obtained from Changchun Yisi experimental animal technology Co., Ltd (Changchun, China). Prior to the experiment, the rats were raised in standardized laboratory conditions: the relative humidity was 40%~60%, the temperature was 20~25 °C with a 12 h light/dark cycle.

After acclimatization for 7 days, the rats were randomly divided into six groups (with 10 in each group): normal group, acetic-acid-induced model group, positive drug (omeprazole, 4.0 mg/kg/day) group, low-dose ocotillol group (5.0 mg/kg/day) (L-ocotillol), moderate-dose ocotillol group (10.0 mg/kg/day) (M-ocotillol), high-dose ocotillol group (20.0 mg/kg/day) (H-ocotillol). The doses were determined based on the pre-experiment. All the rats were fasted for 16 h and then anesthetized with 10% chloral hydrate (3 mL/kg, i.p.), fixed, and a laparotomy was performed. Then, the acetic acid (0.3 mL) was injected under mucosa at the junction of the exposed stomach body and pyloric sinus (except the normal group). The stomach was cautiously put back to the large omentum and the abdomen was sewed 8 Afterwards, the rats were treated with intragastric administration for one week. Normal group and model group were administered 0.9% NaCl aqueous solution (10 mL/kg). The participants in the positive drug group were administered with omeprazole aqueous solution (0.4 mg/mL), ocotillol groups were administered with ocotillol aqueous solution (0.5, 1.0, 2.0 mg/mL).

### 4.4. Preparation of Samples

One hour after the last treatment, the whole blood was collected from abdominal aorta, then clotted at 4 °C for 1 h and centrifuged at 3000 rpm for 10 min at 4 °C to acquire the serum [48] (Lin et al., 2016). A quantity of 1000 µL of the serum was used to measure the levels of ET-1 and NO, and 600 µL of the serum was used to prepare the test sample for metabolomics analysis. The preparation method was as follows: 1800 µL of methanol was added to the serum, then vortex-mixed for 3 min, stood at 4 °C for 10 min, after centrifugation at 10,000 rpm for 10 min at 4 °C, the supernatant was obtained and blew to dryness using a mild stream of nitrogen. The dried residue was dissolved with 200 µL of 80% methanol. After being filtrated with a syringe filter (0.22 µm), the test sample solution was acquired and injected directly into the UPLC system. Meanwhile, a 20 μL aliquot of each test sample solution was mixed to acquire the QC sample for the method validation. 

After the collection of blood, the rats were sacrificed, and the stomach tissues were harvested and washed clean with PBS solution. Gastric mucosa (1 cm×1cm) of each stomach were fixed in 4% formaldehyde for further histological and immunohistochemical analysis. Other gastric mucosa was homogenized with PBS, and the supernatant, for the measurement of EGF, SOD, and NO levels, was obtained by centrifugation at 3,000 rpm for 10 min.

### 4.5. Gastroprotective Effects

#### 4.5.1. Body Weights

The body weights were measured before intragastric administration every day.

#### 4.5.2. ET-1 and NO Levels in Serum

The serum levels of ET-1 and NO were evaluated using ELISA kits, according to the manufacturer’s instructions.

#### 4.5.3. EGF, SOD, and NO Levels in Gastric Mucosa

The levels of EGF, SOD and NO in gastric mucosa were also analyzed using ELISA kits, according to the manufacturer’s instructions.

#### 4.5.4. Histological and Immunohistochemical Analysis

After fixation by 4% formaldehyde, the gastric mucosa was dehydrated with gradient alcohol and embedded in paraffin. Sections of 5 μm intervals were stained with hematoxylin and eosin (H&E) and observed for pathological changes. In addition, immuno-histochemical staining was also applied to evaluate the ET-1, NOS2, and EGFR levels.

#### 4.5.5. Molecular Docking

The molecular docking was used to calculate the relative binding free energies and the localized binding sites in the active pocket. In order to illuminate the action mode of ocotillol on EGF and NOS2, a molecular docking study was carried out by using Grid-based Ligand Docking with Energetics (GLIDE, Schrödinger, New York, USA, Version 2015) software. The main steps include protein preparation, ligand preparation, receptor grid generation and glide docking.

After being retrieved from the Protein Data Bank (PDB) database (http://www.rcsb.org/pdb), the X-ray crystal structures of EGF (PDB code: 3RCD) and NOS2 (PDB code: 3EAI) were converted to Maestro files [51,52,53,54] by PDB conversion library. Then, the structures were further optimized by assigning water orientations and bond orders, removing water, adding hydrogen, and creating zero-order bonds to metals and di-sulphide bonds. A two-dimensional structure of ocotillol was drawn by Maestro Elements (Maestro Elements, Version 2.2, Schrödinger, New York, USA, Version 2015) and its three-dimensional structure was generated using the LigPrep module of Schrödinger Suite. The prepared structures of EGF, NOS2 and ocotillol were imported into the workspace for GLIDE docking.

Extra-precision docking was performed, and the default values of scaling factor and partial charge cutoff were set at 0.80 and 0.15, respectively. Finally, PyMOL (Schrödinger) was used to generate the figures of the docking results.

### 4.6. Data Analysis

The results were expressed as mean ± standard deviation (SD). Statistical analysis was performed using one-way analysis of variance (ANOVA) and Tukey’s test. Statistical significance was set as * *p* < 0.05, and high statistical significance was set as ** *p* < 0.01.

### 4.7. Metabolomics Study

MarkerLynx XS Version 4.1 software (Waters Co., Milford, MA, USA.) was used to control the system, execute the sample list and acquire raw data. The major processing parameters were set as follows: mass range 50~1200 Da, mass window 0.10, mass tolerance 0.10, retention time range 2~28 min, retention time window 0.20, marker intensity threshold 2000 counts and noise elimination level 6. Thus, the exact mass/retention time pairs and their corresponding intensities of all peaks were shown in Extended Statistics (XS) Viewer. Then, the exported data were imported to SIMCA-P sofware (Version 14.1, Umetric, Umea, Sweden) for performing multivariate analysis including principle component analysis (PCA) and orthogonal projections to latent structures discriminant analysis (OPLS-DA). PCA, an unsupervised method of pattern recognition approach, could obtain the overview and classification showing maximum variation and pattern recognition. OPLS-DA was used to obtain the maximum separation between two different groups. S-plots, which could provide visualization of the OPLS-DA predictive results, were created to explore the potential biomarkers that made a remarkable contribution to the metabolic distinction. Meanwhile, metabolites with the variable importance in the projection (VIP) value above 1.0 and p-value below 0.05 were considered as potential biomarkers. Furthermore, a permutation test was also performed to provide a reference distribution with the R2/Q2 values to indicate statistical significance. The predictive receiver operating characteristic (ROC) curves were generated using the metabolites identified with the area under curve (AUC) > 0.8 and *p* < 0.01. The potential biomarkers were then discovered.

Afterwards, several biochemical databases including METLIN (http://metlin.scripps.edu/), Metabo-Analyst (http://www.metaboanalyst.ca/), HMDB (http://www.hmdb.ca/) and KEGG (http://www.kegg.com/) were applied to confirm the biomarkers. The biomarkers were further identified by either referring the chemical standards or comparing the tandem mass spectrometry (MS/MS) fragmentation patterns according to HMDB and METLIN databases. The adducts were [M+H]+ and [M+Na]+ in ESI+, [M-H]− and [M+FA-H]− in ESI−, with the mass tolerance at 10 ppm. Then, the MetaboAnalyst 4.0 was used to analyse the confirmed distinct metabolites to filter out the most vital potential metabolic pathways, with the impact-value threshold above 0.10.

## 5. Conclusions

Taken together, the results of our study show that ocotillol had a protective effect in an acetic-acid-induced rat GU model through the regulation of relevant metabolic pathways, such as caffeine metabolism, sphingolipid metabolism, arachidonic acid metabolism, linoleic acid metabolism, glycerophospholipid metabolism, retinol metabolism and ether lipid metabolism. This study helps us to understand the pathogenesis of GU and to provide a potential natural anti-ulcer agent.

## Figures and Tables

**Table 1:** Distinct metabolites identified in serum samples.

No.	RT	Mass	Compound Name	VIP	Formula	Adducts	Δm	HMDB ID	Pathways	Content Level
1*	0.60	203.0532	Paraxanthine	2.77	C_7_H_8_N_4_O_2_	M+Na	0	HMDB0001860	CM [22]	C_M_ < C_D_
2^a^	11.39	972.7340	Galabiosylceramide (d18:1/24:1(15Z))	1.53	C_54_H_101_NO_13_	M+H	−1.13	HMDB0004837	SphM [23]	C_M_ < C_D_
3^a^	11.40	846.6355	PC(22:2(13Z,16Z)/P-18:1(11Z))	2.62	C_48_H_90_NO_7_P	M+Na	0.24	HMDB0008621	AM, LM, GlyM [24,25,26]	C_D_ < C_M_
4^a^	11.49	890.6626	PC(18:2(9Z,12Z)/24:1(15Z))	1.99	C_50_H_94_NO_8_P	M+Na	1.24	HMDB0008158	AM, LM,GlyM [24,25,26]	C_D_ < C_M_
5*	12.72	318.3010	Phytosphingosine	6.98	C_18_H_39_NO_3_	M+H	0.63	HMDB0004610	SphM [25,27]	C_M_<C_D_
6*	14.94	302.3054	Sphinganine	2.95	C_18_H_39_NO_2_	M+H	−1.65	HMDB0000269	SphM [25,27]	C_M_<C_D_
7^a^	16.90	544.3398	LysoPC(18:1(9Z))	7.92	C_26_H_52_NO_7_P	M+Na	3.49	HMDB0002815	GlyM [24,25]	C_D_<C_M_
8^a^	18.04	303.2326	Retinyl ester	1.91	C_20_H_30_O_2_	M+H	0.66	HMDB0003598	RM [28]	C_M_<C_D_
9^a^	18.07	814.5580	PC(15:0/20:3(5Z,8Z,11Z))	1.09	C_43_H_80_NO_8_P	M+FA-H	−2.21	HMDB0007947	AM, LM,GlyM [24,25,26]	C_D_<C_M_
10*	18.11	319.2276	19(S)-HETE	7.39	C_20_H_32_O_3_	M-H	0.94	HMDB0011136	AM [29]	C_D_<C_M_
11^a^	20.54	524.3709	PC(O-16:0/2:0)	18.53	C_26_H_54_NO_7_P	M+H	−1.33	HMDB0062195	EM [30]	C_D_<C_M_
12^a^	20.90	550.3883	PC(18:1(9Z)e/2:0)	2.10	C_28_H_56_NO_7_P	M+H	1.82	HMDB0011148	AM, LM,GlyM [24,25,26]	C_D_<C_M_
13^a^	21.15	510.3917	LysoPC(O-18:0/0:0)	1.76	C_26_H_56_NO_6_P	M+H	−1.37	HMDB0011149	EM [31]	C_D_<C_M_
14*	22.81	303.2327	Arachidonic acid	7.16	C_20_H_32_O_2_	M-H	0.99	HMDB0001043	AM [24]	C_D_<C_M_
15*	23.13	279.2325	Linoleic acid	1.40	C_18_H_32_O_2_	M-H	0.36	HMDB0000673	LM [28]	C_M_<C_D_
16^a^	24.68	806.5672	PC(14:1(9Z)/22:2(13Z,16Z))	1.87	C_44_H_82_NO_8_P	M+Na	−0.50	HMDB0007921	AM, LM,GlyM [24,25,26]	C_D_<C_M_
17^a^	26.33	786.6015	PC(18:2(9Z,12Z)/18:0)	5.38	C_44_H_84_NO_8_P	M+H	0.25	HMDB0008135	AM, LM,GlyM [24,25,26]	C_M_<C_D_
18^a^	27.41	703.5740	SM(d18:1/16:0)	6.72	C_39_H_80_N_2_O_6_P	M+H	−1.99	HMDB0010169	SphM [27]	C_M_<C_D_
19^a^	27.54	782.5677	PC(14:0/20:1(11Z))	3.68	C_42_H_82_NO_8_P	M+Na	0.13	HMDB0007879	AM, LM,GlyM [24,25,26]	C_D_<C_M_
20^a^	27.67	826.5592	PC(14:0/22:4(7Z,10Z,13Z,16Z))	1.50	C_44_H_80_NO_8_P	M+FA-H	−0.73	HMDB0007889	AM, LM,GlyM [24,25,26]	C_D_<C_M_
21^a^	27.91	802.5610	PC(14:0/20:2(11Z,14Z))	2.76	C_42_H_80_NO_8_P	M+FA-H	1.50	HMDB0007880	AM, LM,GlyM [24,25,26]	C_D_<C_M_

RT, Retention Time, min; Mass, Measured mass, Da; Δm, Relative Deviation, ppm; * Metabolites validated with standards; ^a^ Metabolites confirmed by MS/MS fragments; “D” represents drug intervention group (H-ocotillol group); “M” represents model group; “PC”: Phosphatidylcholine; “LysoPC”: Lysophosphatidylcholine; “HETE”: Hydroxyeicosatetraenoic acid; SM: Sphingomyelin.

**Table 2:** The area under curve (AUC) values and p values of the biomarkers in two predictive ROC curves.

No.	M and N		M and Ocotillol	
	AUC	*p*	AUC	*p*
1	1.000	<0.001	1.000	<0.001
2	1.000	<0.001	1.000	<0.001
3	1.000	<0.001	1.000	<0.001
4	0.960	0.0001	0.990	<0.001
5	1.000	<0.001	1.000	<0.001
6	1.000	<0.001	1.000	<0.001
7	1.000	<0.001	1.000	<0.001
8	0.900	0.002	0.960	0.001
9	0.860	0.007	1.000	<0.001
10	0.990	<0.001	0.990	<0.001
11	1.000	<0.001	1.000	<0.001
12	0.980	<0.001	0.990	<0.001
13	1.000	<0.001	1.000	<0.001
14	1.000	<0.001	1.000	<0.001
15	0.950	0.001	0.940	0.001
16	1.000	<0.001	1.000	<0.001
17	1.000	<0.001	1.000	<0.001
18	0.980	<0.001	0.990	<0.001
19	1.000	<0.001	1.000	<0.001
20	0.910	0.002	0.990	<0.001
21	0.980	<0.001	0.930	0.001

**Table 3:** The results from metabolic pathways of differential metabolites.

Pathway Name	Match Status	*p*	−log (*p*)	Holm *p*	FDR	Impact
Sphingolipid metabolism (SphM)	4/21	1.8325 × 10^−5^	10.9070	0.0015	0.0015	0.1968
Linoleic acid metabolism (LM)	2/5	6.7558 × 10^−4^	7.2999	0.0560	0.0284	1.0000
Arachidonic acid metabolism (AM)	3/36	0.0030	5.7997	0.2484	0.0848	0.3329
Ether lipid metabolism (EM)	2/20	0.0119	4.4286	0.9664	0.2506	0.2289
Glycerophospholipid metabolism (GlyM)	2/36	0.0366	3.3077	1.0000	0.5124	0.1118
Caffeine metabolism (CM)	1/12	0.0990	2.3130	1.0000	1.0000	0.6923
Retinol metabolism (RM)	1/16	0.1299	2.0411	1.0000	1.0000	0.1617

## Supplementary Materials

Supplementary materials can be found at https://www.mdpi.com/1422-0067/21/7/2577/s1.

## Abbreviations

ANOVA	Analysis of variance
AUC	Area under curve
EGF	Epidermal growth factor
EGFR	Epidermal growth factor receptor
ELISA	Enzymeelinked immunosorbent assay
ESI	Electrospray ionization
ET-1	Endothelin-1
GU	Gastric ulcer
H&E	Hematoxylin and eosin
HETE	Hydroxyeicosatetraenoic acid
H-ocotillol	high-dose ocotillol
L-ocotillol	low-dose ocotillol
LysoPC	Lysophosphatidylcholine
M-ocotillol	moderate-dose ocotillol
NOS2	Inducible nitric oxide synthase
OPLS-DA	Orthogonal Projections to Latent Structures Discriminant Analysis
PC	Phosphatidylcholine
PCA	Principal Component Analysis
PDB	Protein Data Bank
QC	Quality Control
QTOF-MS	Quadrupole Time of Flight-Mass Spectrometry
ROC	Receiver operating characteristic
RSD	Relative Standard Deviation
RT	Retention Time
SD	standard deviation
SM	Sphingomyelin
SOD	Superoxide dismutase
UPLC	Ultra-Performance Liquid Chromatography
VIP	Variable importance in the projection
