# Supplementary material for: Protective Effect of Ocotillol, the Derivate of Ocotillol-Type Saponins in Panax Genus, against Acetic Acid-Induced Gastric Ulcer in Rats Based on Untargeted Metabolomics
Source: Int J Mol Sci. 2020 Apr 8;21(7):2577. doi: 10.3390/ijms21072577 (PMC7177626; doi:10.3390/ijms21072577)
Supplement: Supplementary file 1 [file ijms-21-02577-s001.pdf]

Supplementary Material

PART I: MS/MS spectra of *standards* and *identified potential markers*

There were six of the putative markers were confirmed with available reference standards by matching their retention time and accurate mass measurement. The MS/MS spectra of each standard and corresponding identified potential marker are shown in Figure S1-6.

A

B

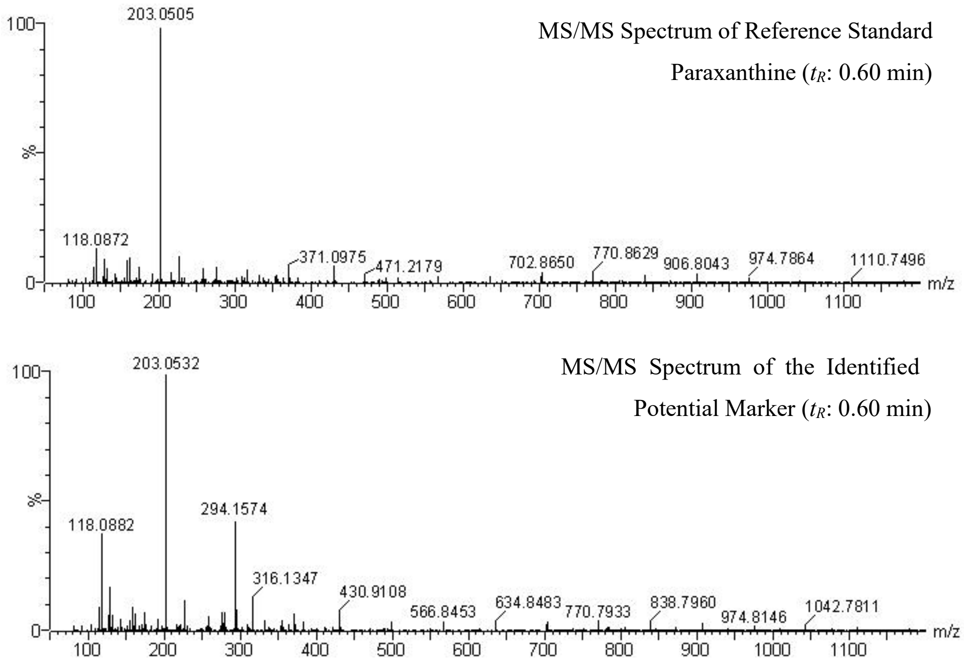

Figure S1. The MS/MS spectra of Paraxanthine (A) and identified potential marker (B

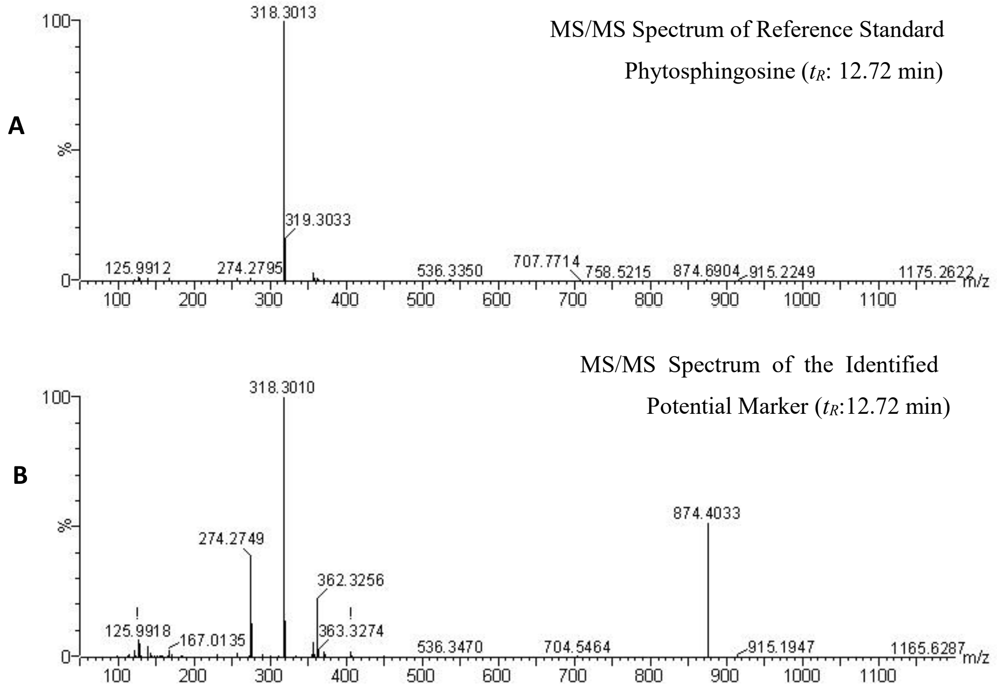

Figure S2. The MS/MS spectra of Phytosphingosine (A) and identified potential marker (B)

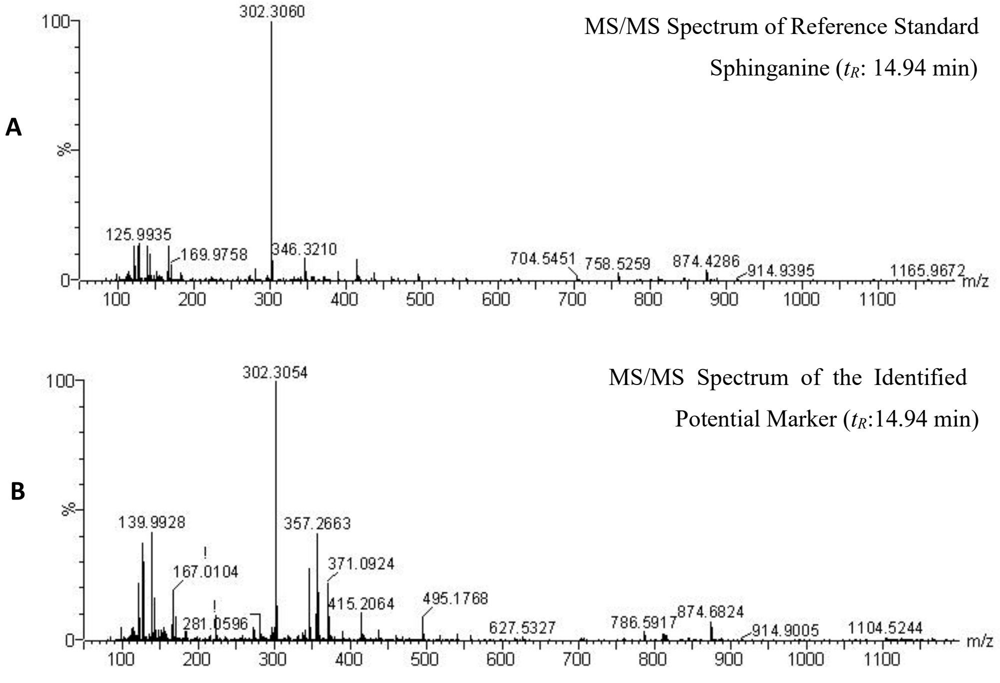

Figure S3. The MS/MS spectra of Sphinganine (A) and identified potential marker (B)

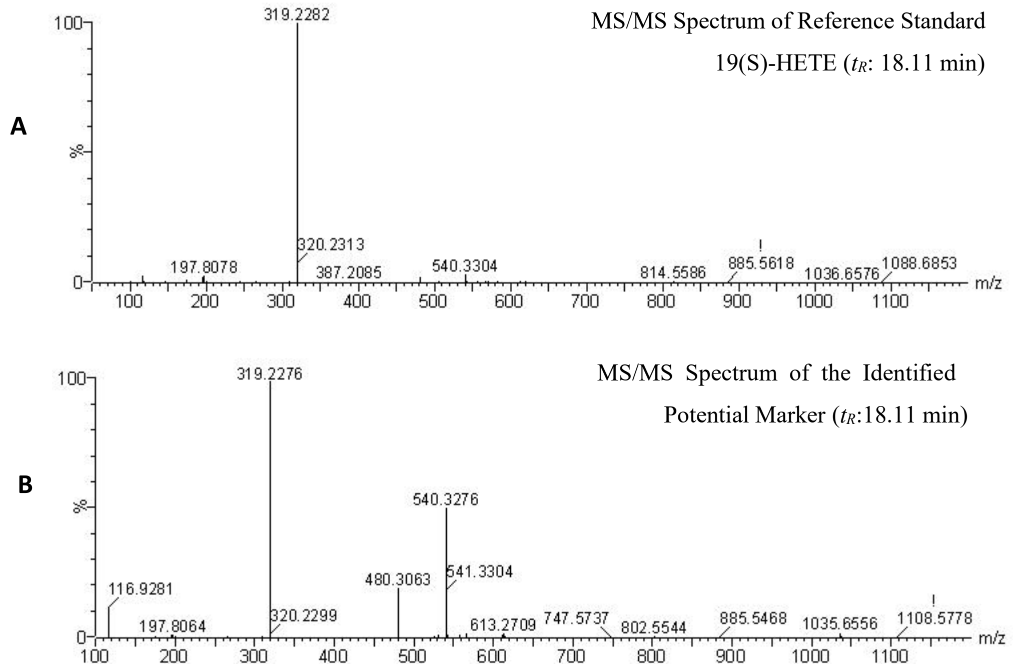

Figure S4. The MS/MS spectra of 19(S)-HETE (A) and identified potential marker (B)

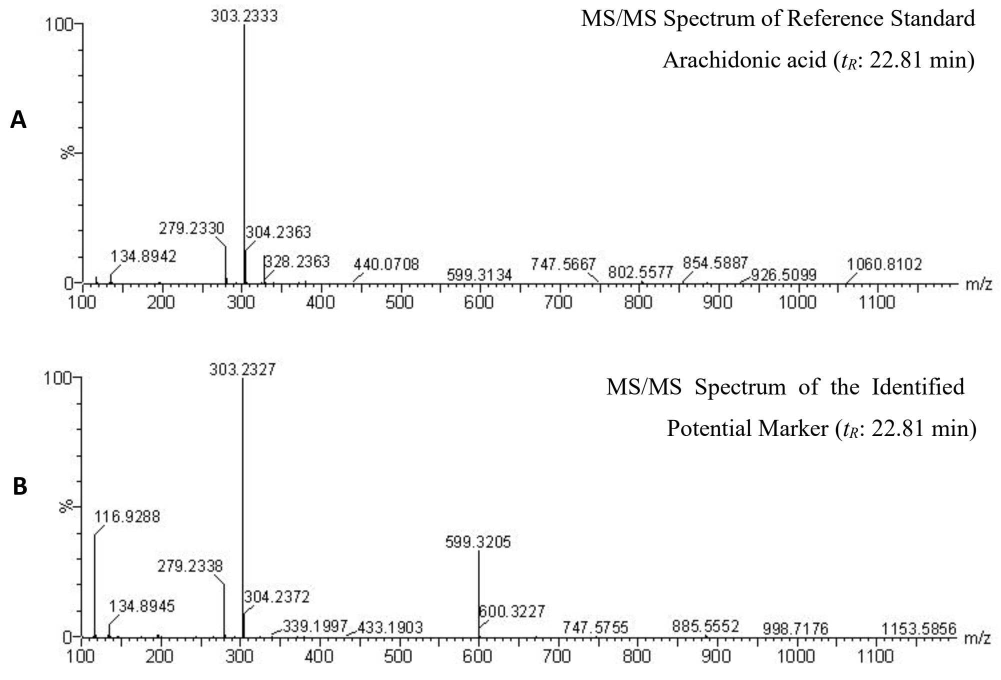

Figure S5. The MS/MS spectra of Arachidonic acid(A) and identified potential marker (B)

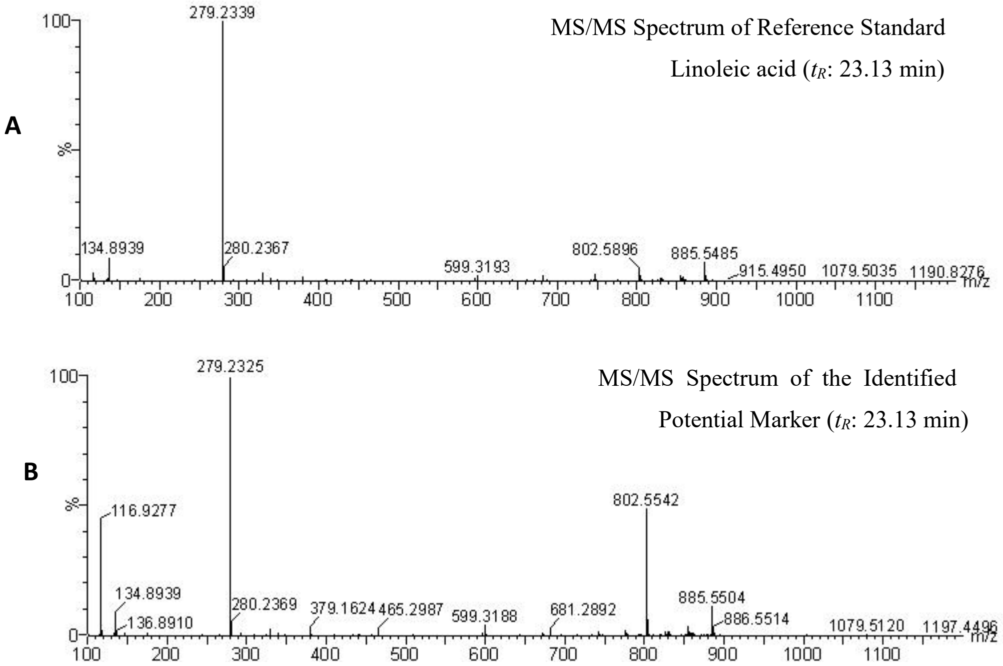

Figure S6. The MS/MS spectra of Linoleic acid(A) and identified potential marker (B)

PART II: MS/MS spectra of the identified potential markers by comparing accurate molecular weight and tandem mass spectrometry

There were other fifteen putative structures of the metabolites were identified by comparing accurate molecular weight and tandem mass spectrometry obtained in the study with the information recorded in biochemical database (HMDB) or Metlin, or analyzing their MS/MS fragments. The MS/MS spectra of each identified potential marker and corresponding information are shown in Figure S7-21.

MS/MS Spectrum of identified potential marker Galabiosylceramide (d18:1/24:1(15Z)) (t_R: 11.39 min)

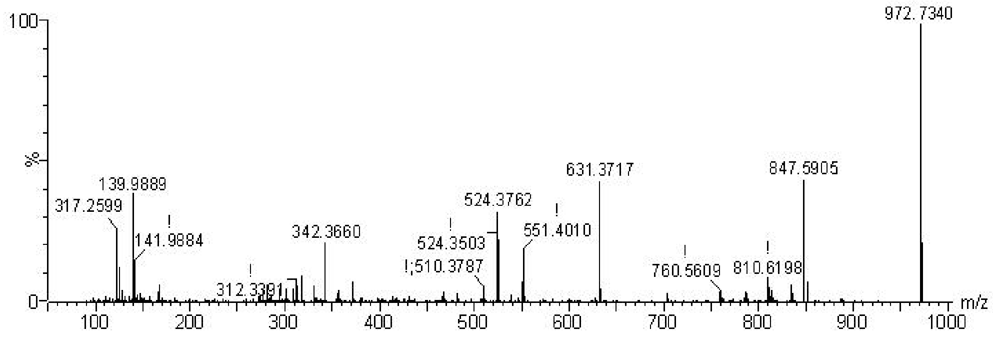

Figure S7. The MS/MS spectra of identified potential marker Galabiosylceramide (d18:1/24:1(15Z))

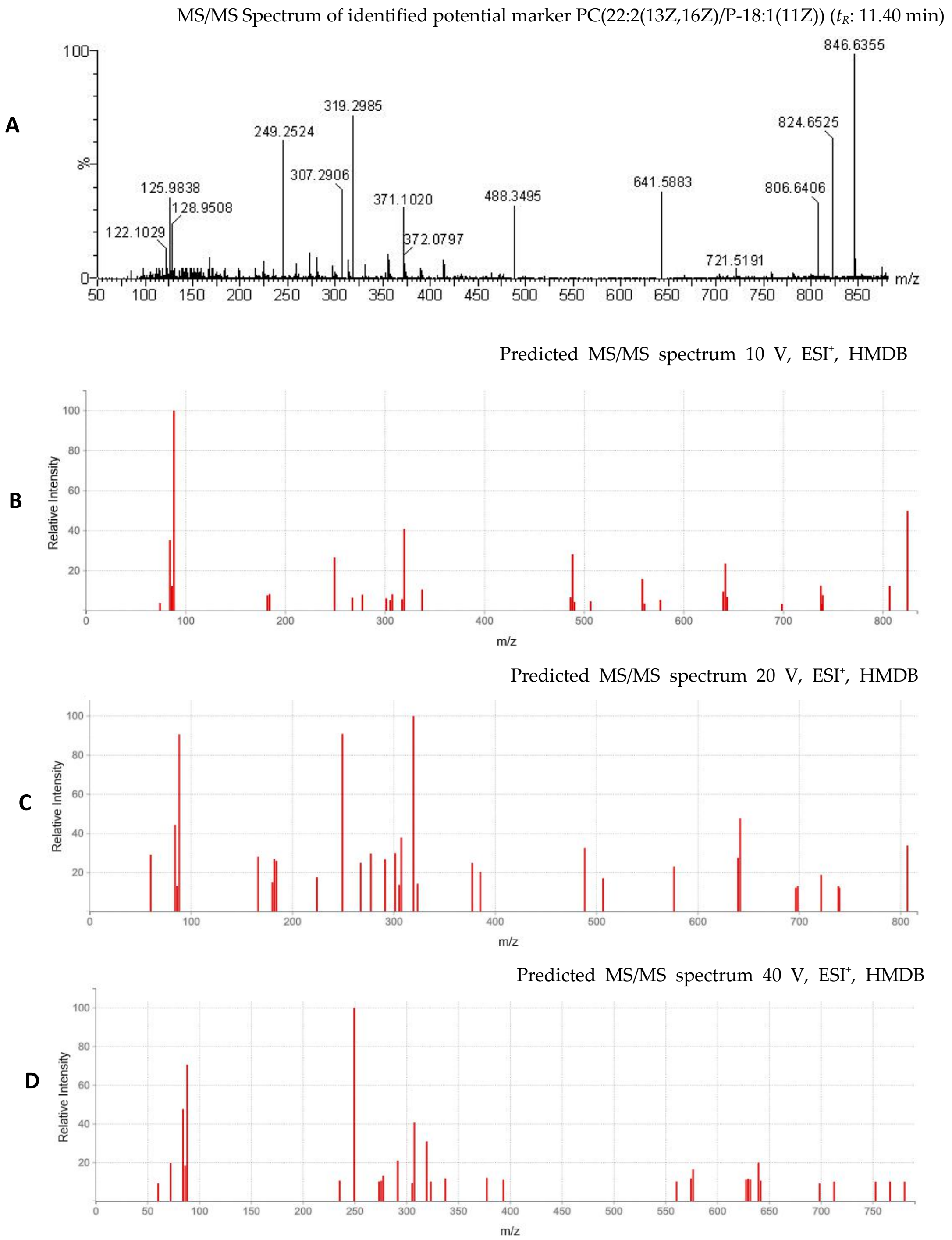

Figure S8. The MS/MS spectra of identified potential marker PC(22:2(13Z,16Z)/P-18:1(11Z)) (A) and the MS/MS spectrum in HMDB database (B, C, D)

MS/MS Spectrum of identified potential marker PC(18:2(9Z,12Z)/24:1(15Z)) (t_R: 11.49 min)

A

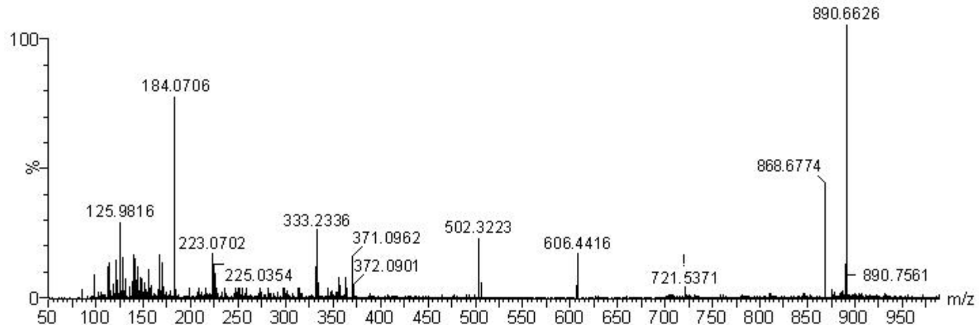

Predicted MS/MS spectrum 10 V, ESI$^+$, HMDB

B

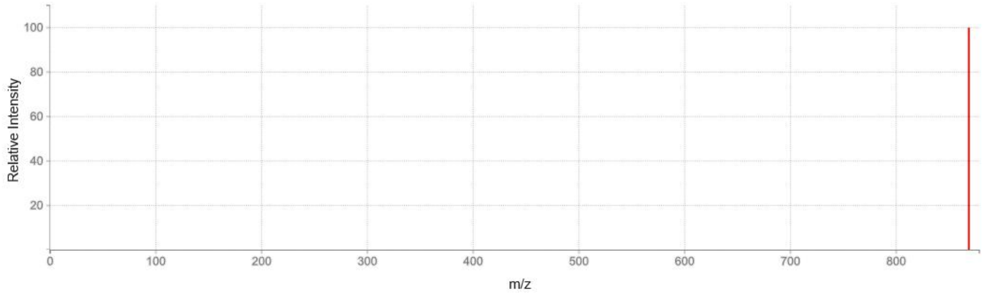

Predicted MS/MS spectrum 20 V, ESI$^+$, HMDB

C

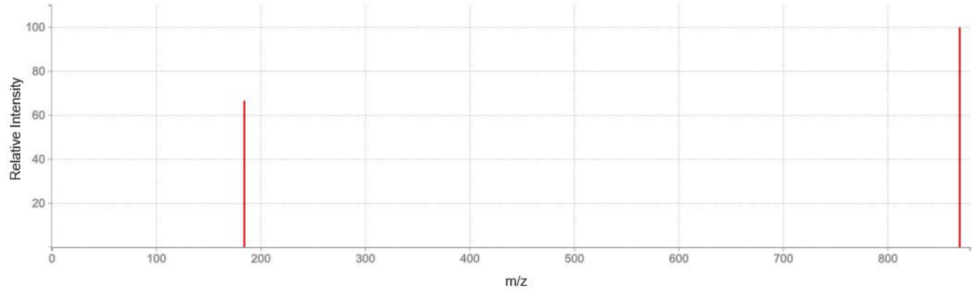

Predicted MS/MS spectrum 40 V, ESI$^+$, HMDB

D

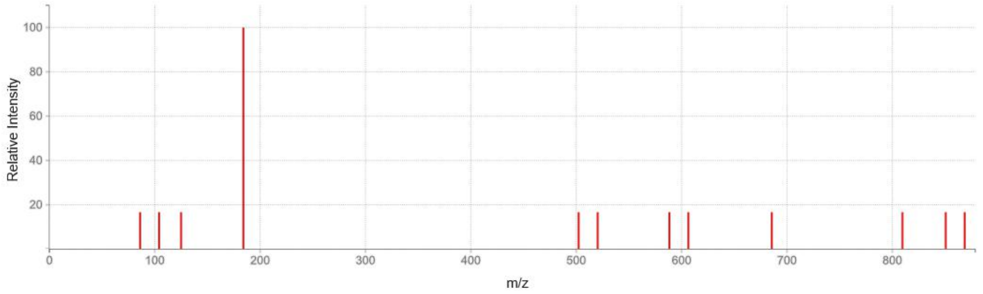

Figure S9. The MS/MS spectra of identified potential marker PC(18:2(9Z,12Z)/24:1(15Z)) (A) and the MS/MS spectrum in HMDB database (B, C, D)

MS/MS Spectrum of identified potential marker LysoPC(18:1(9Z)) (t_R: 16.90 min)

A

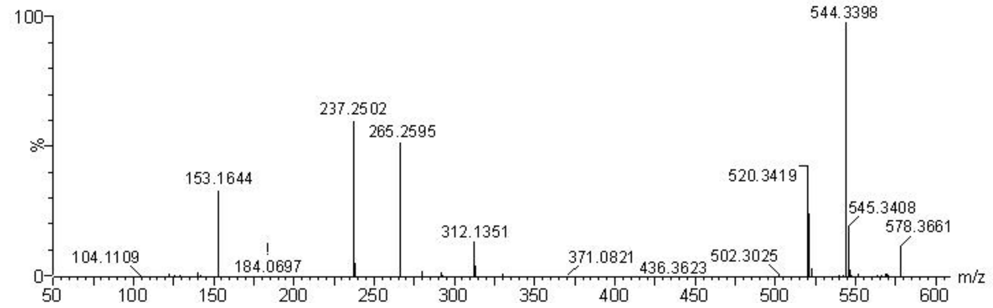

Predicted MS/MS spectrum 70 V, ESI$^+$, HMDB

B

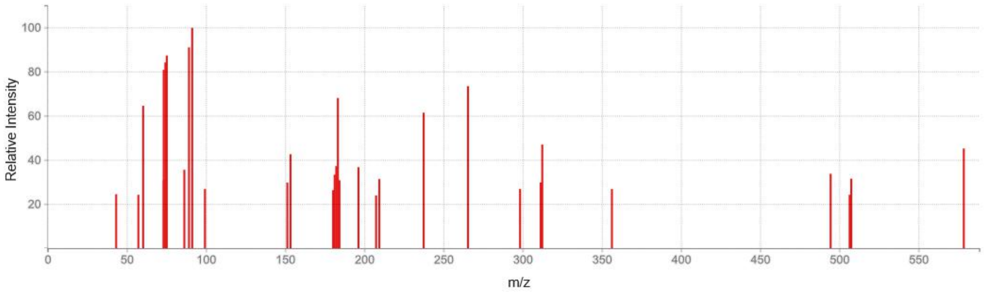

Figure S10. The MS/MS spectra of identified potential marker LysoPC(18:1(9Z)) (A) and the MS/MS spectrum in HMDB database (B)

MS/MS Spectrum of identified potential marker Retinyl ester (t_R: 18.04 min)

A

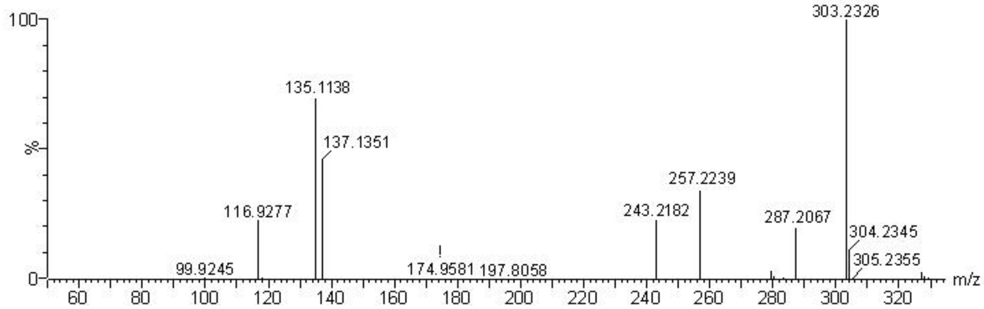

Predicted MS/MS spectrum 10 V, ESI⁺, HMDB

B

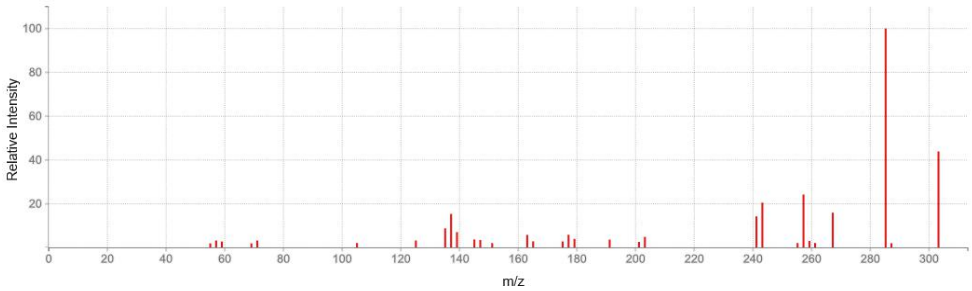

Predicted MS/MS spectrum 20 V, ESI⁺, HMDB

C

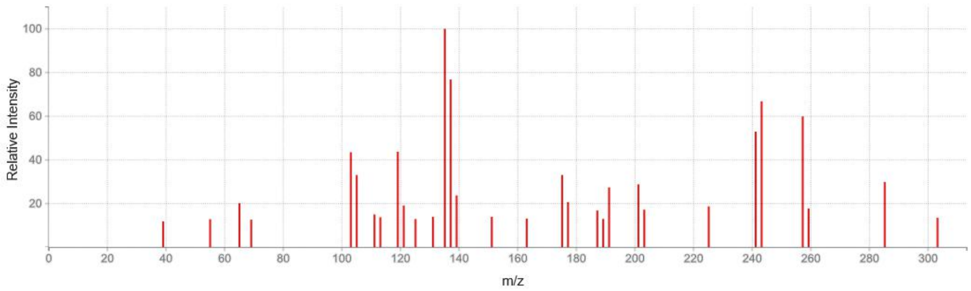

Predicted MS/MS spectrum 40 V, ESI⁺, HMDB

D

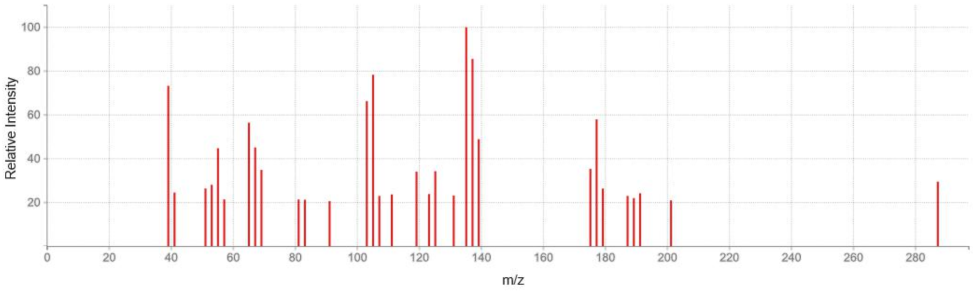

Figure S11. The MS/MS spectra of identified potential marker LysoPC(18:1(9Z)) (A) and the MS/MS spectrum in HMDB database (B, C, D)

MS/MS Spectrum of identified potential marker PC(15:0/20:3(5Z,8Z,11Z)) (t_R: 18.07 min)

A

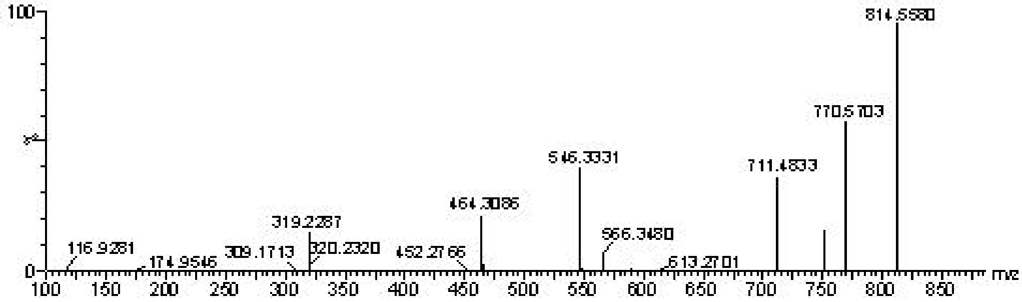

Predicted MS/MS spectrum 10 V, ESI$^-$, HMDB

B

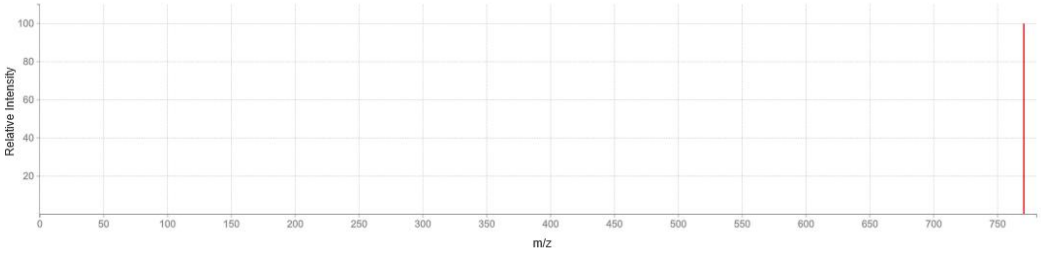

Predicted MS/MS spectrum 20 V, ESI$^-$, HMDB

C

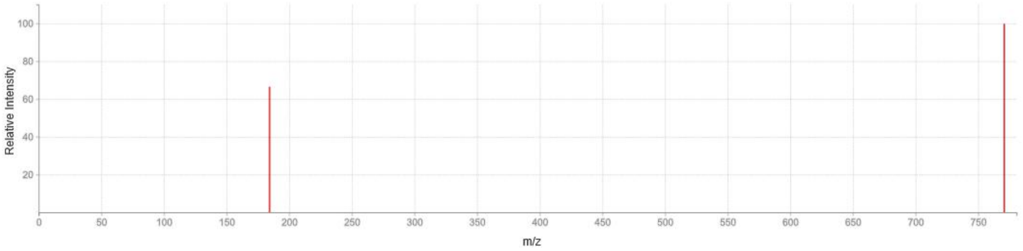

Predicted MS/MS spectrum 40 V, ESI$^-$, HMDB

D

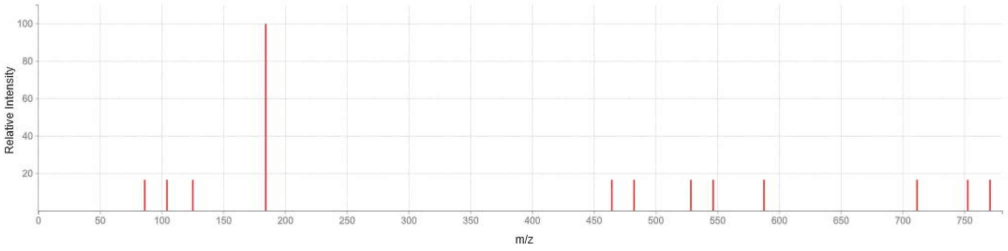

Figure S12. The MS/MS spectra of identified potential marker PC(15:0/20:3(5Z,8Z,11Z)) (A) and the MS/MS spectrum in HMDB database (B, C, D)

MS/MS Spectrum of identified potential marker PC(O-16:0/2:0) (t_R: 20.54 min)

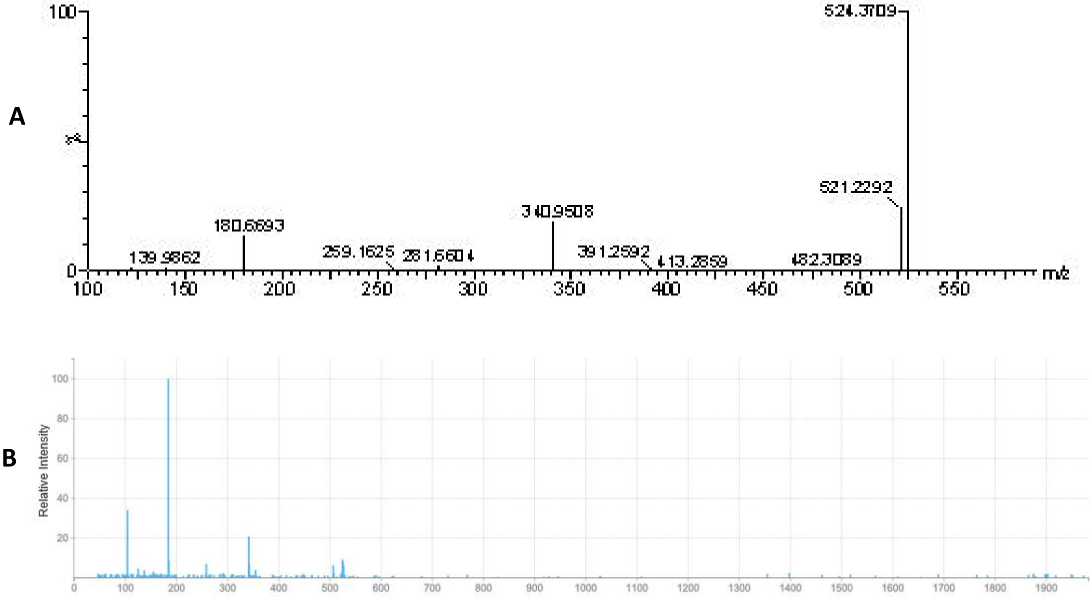

Figure S13. The MS/MS spectra of identified potential marker PC(O-16:0/2:0)(A) and the MS/MS spectrum in HMDB database (B)

MS/MS Spectrum of identified potential marker PC(18:1(9Z)e/2:0) (t_R: 20.90 min)

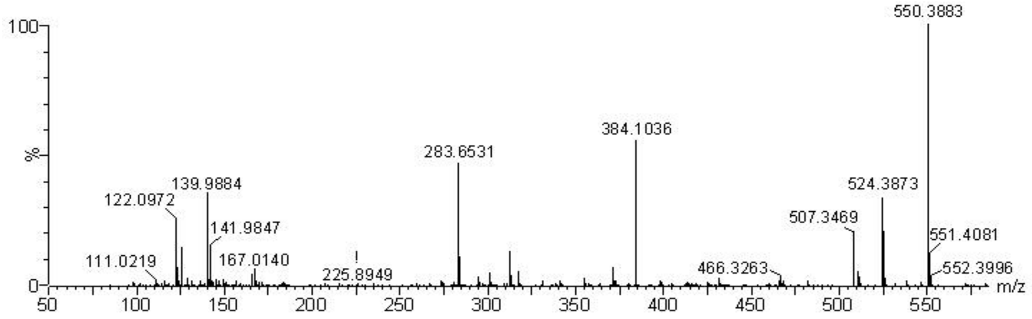

Figure S14. The MS/MS spectra of identified potential marker PC(18:1(9Z)e/2:0)

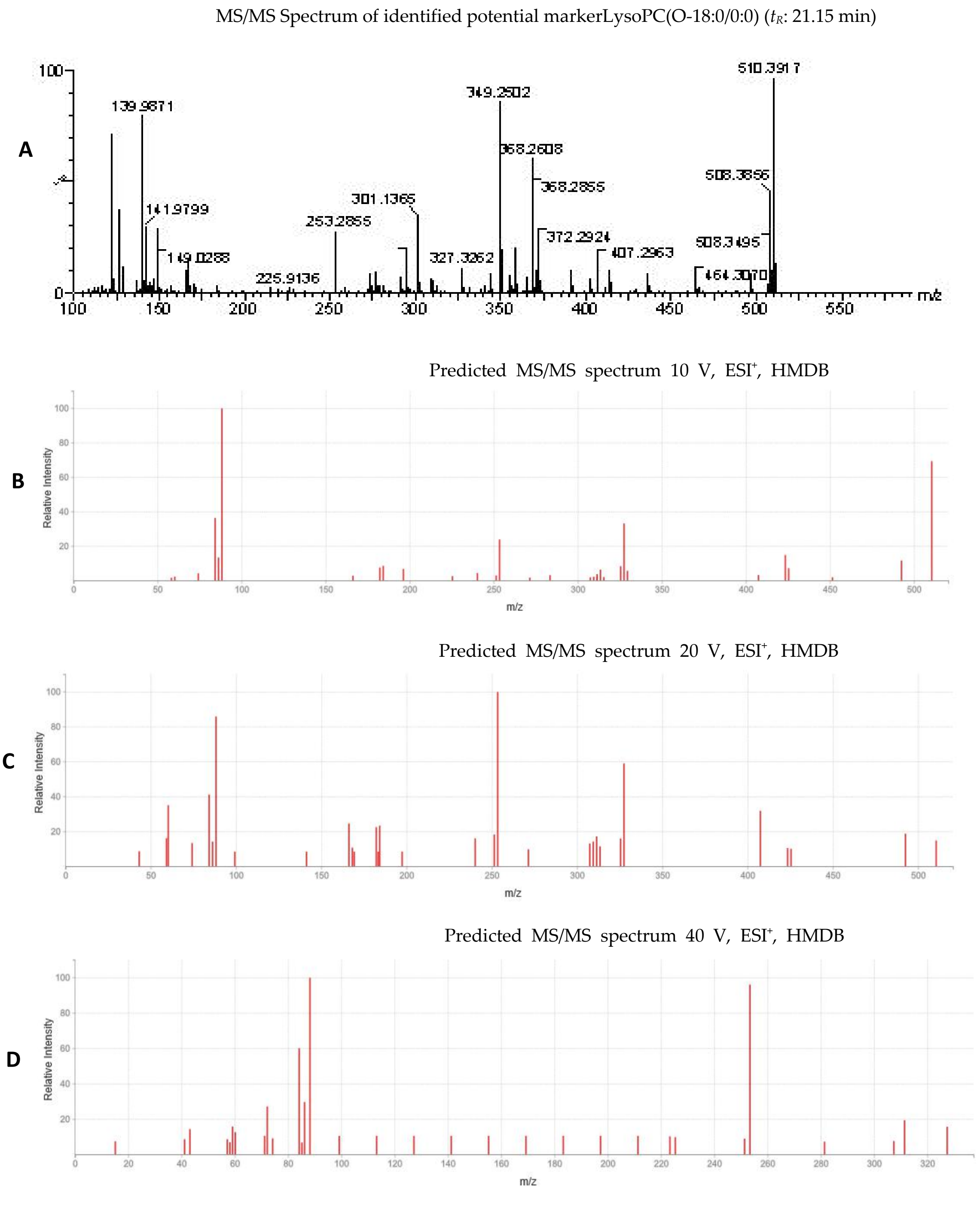

Figure S15. The MS/MS spectra of identified potential marker LysoPC(O-18:0/0:0) (A) and the MS/MS spectrum in HMDB database (B, C, D)

MS/MS Spectrum of identified potential marker PC(14:1(9Z)/22:2(13Z,16Z)) (t_R: 24.68 min)

A

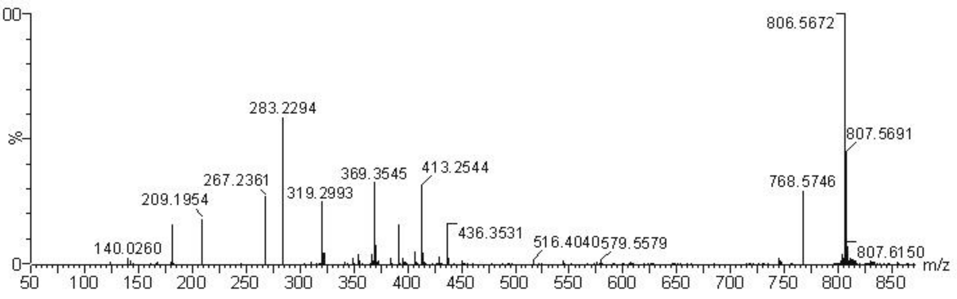

Predicted MS/MS spectrum 10 V, ESI⁺, HMDB

B

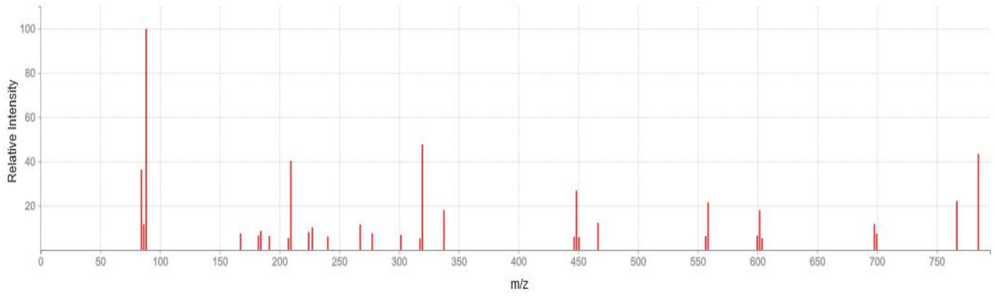

Predicted MS/MS spectrum 20 V, ESI⁺, HMDB

C

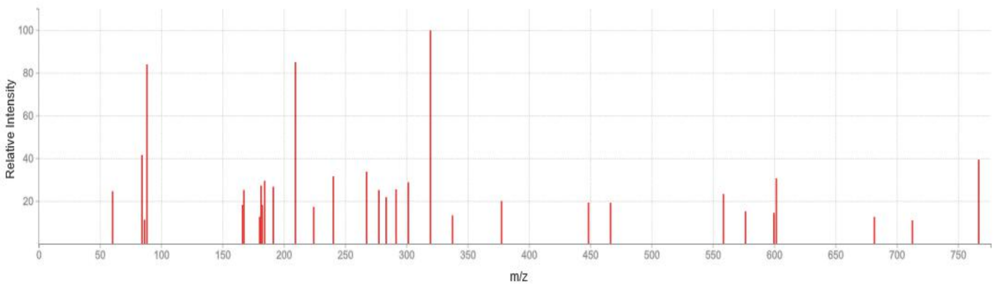

Predicted MS/MS spectrum 40 V, ESI⁺, HMDB

D

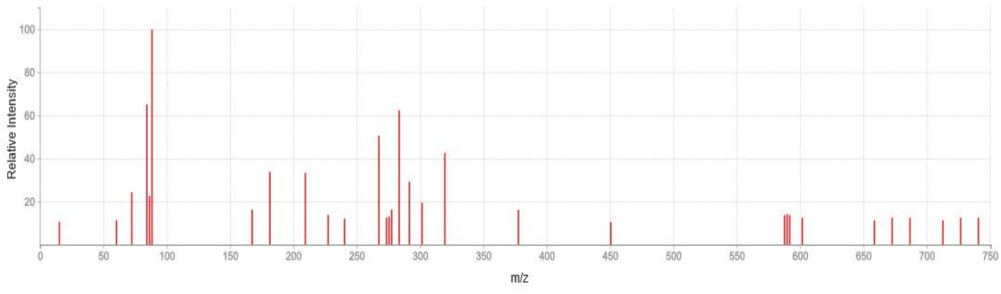

Figure S16. The MS/MS spectra of identified potential marker PC(14:1(9Z)/22:2(13Z,16Z)) (A) and the MS/MS spectrum in HMDB database (B, C, D)

MS/MS Spectrum of identified potential marker PC(18:2(9Z,12Z)/18:0) (t_R: 26.33 min)

A

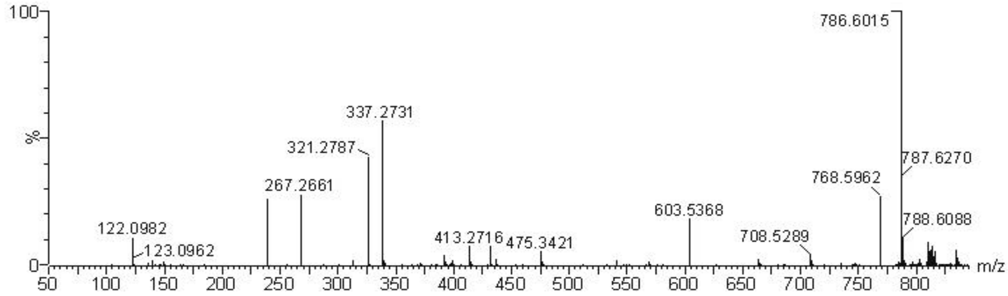

Predicted MS/MS spectrum 10 V, ESI$^+$, HMDB

B

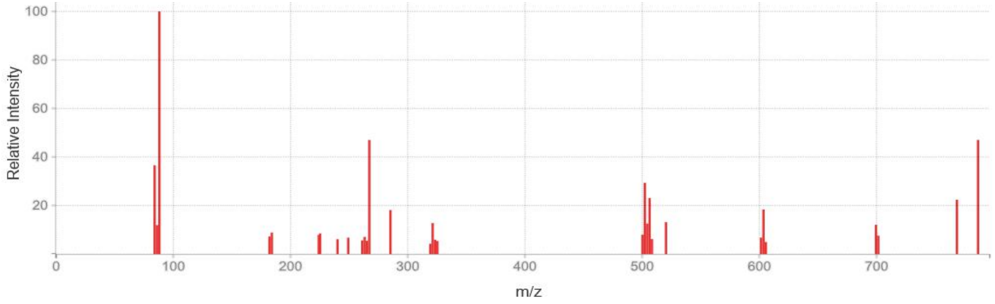

Predicted MS/MS spectrum 20 V, ESI$^+$, HMDB

C

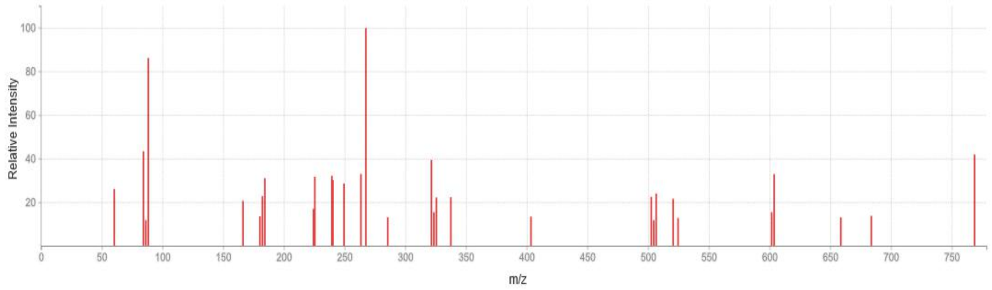

Predicted MS/MS spectrum 40 V, ESI$^+$, HMDB

D

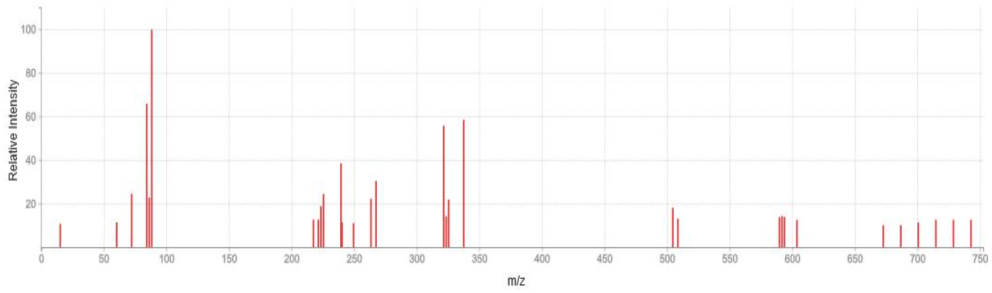

Figure S17. The MS/MS spectra of identified potential marker PC(18:2(9Z,12Z)/18:0) (A) and the MS/MS spectrum in HMDB database (B, C, D)

MS/MS Spectrum of identified potential marker SM(d18:1/16:0) (t_R: 27.41 min)

A

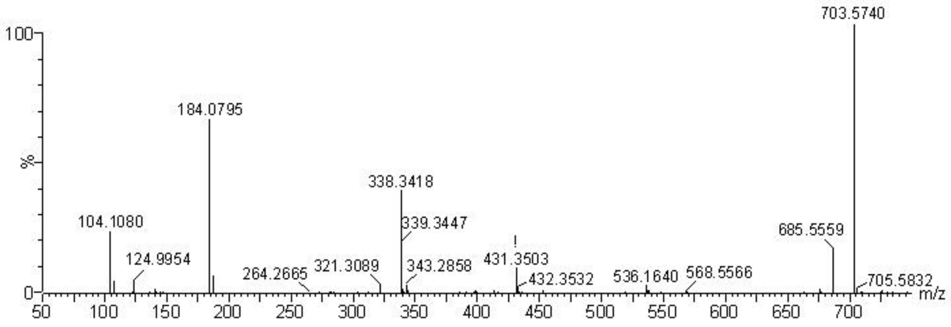

Predicted MS/MS spectrum 10 V, ESI⁺, HMDB

B

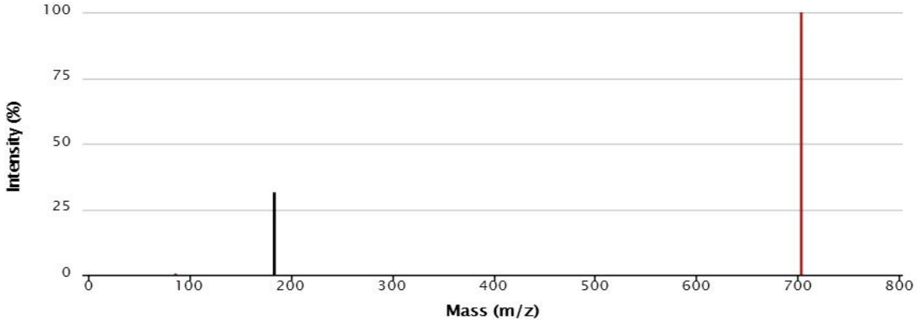

Predicted MS/MS spectrum 20 V, ESI⁺, HMDB

C

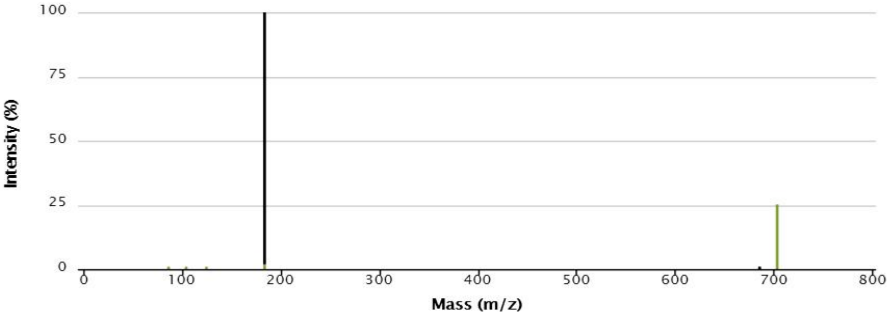

Predicted MS/MS spectrum 40 V, ESI⁺, HMDB

D

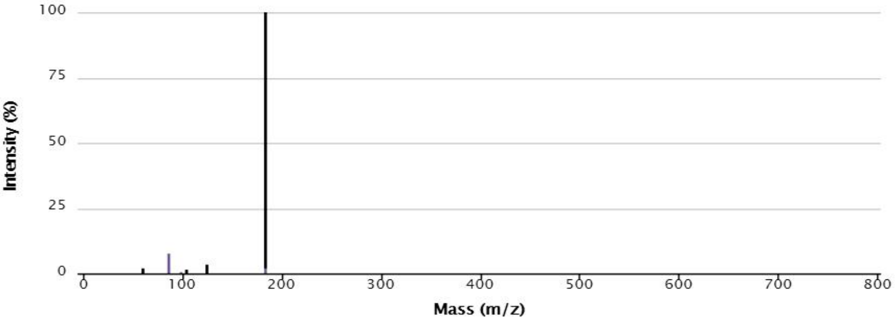

Figure S18. The MS/MS spectra of identified potential marker SM(d18:1/16:0) (A) and the MS/MS spectrum in Metlin database (B, C, D)

MS/MS Spectrum of identified potential marker PC(14:0/20:1(11Z)) (t_R: 27.54 min)

A

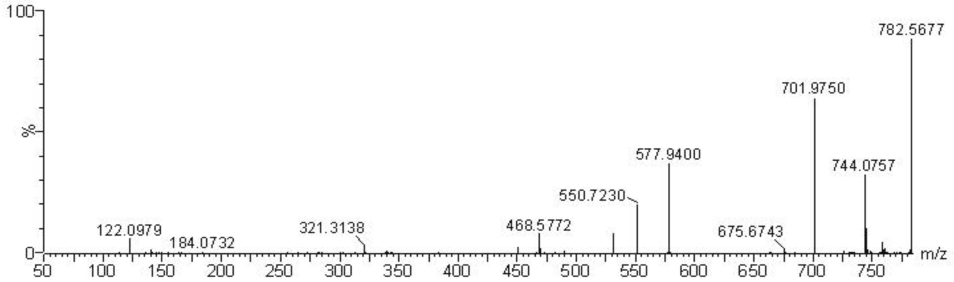

Predicted MS/MS spectrum 10 V, ESI$^+$, HMDB

B

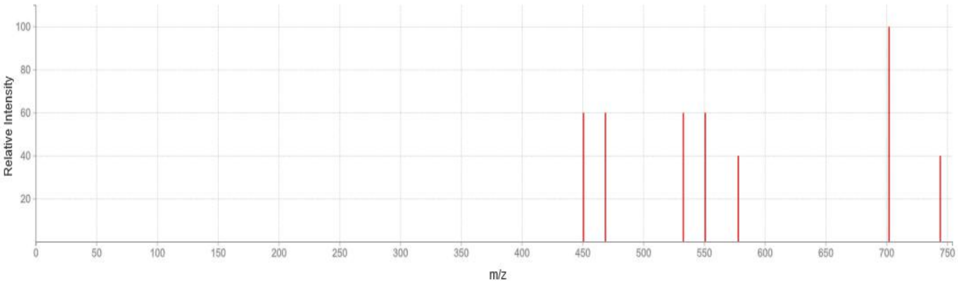

Figure S19. The MS/MS spectra of identified potential marker PC(14:0/20:1(11Z)) (A) and the MS/MS spectrum in HMDB database (B, C, D)

MS/MS Spectrum of identified potential marker PC(14:0/22:4(7Z,10Z,13Z,16Z)) (t_R: 27.67 min)

A

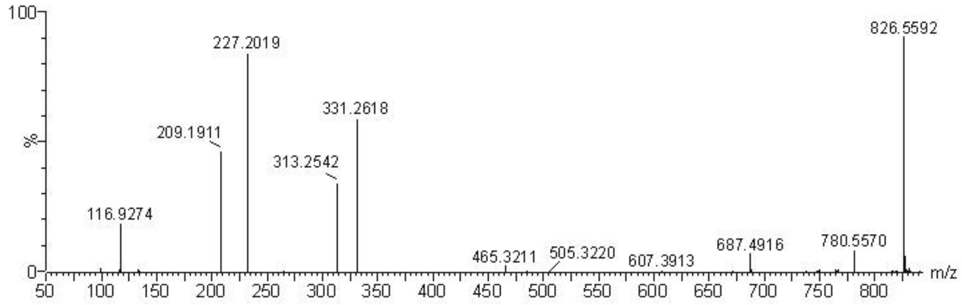

Predicted MS/MS spectrum 10 V, ESI⁻, HMDB

B

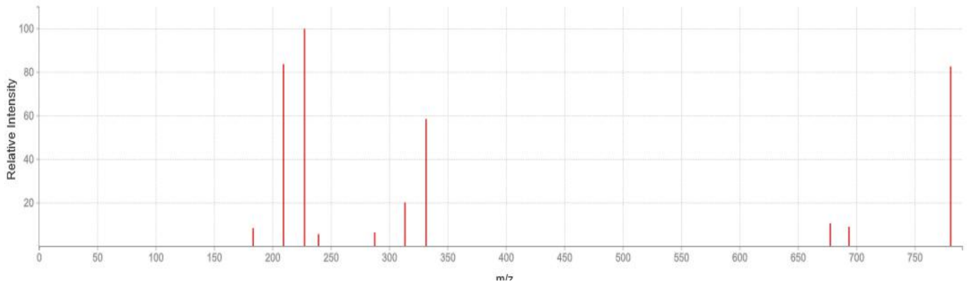

Predicted MS/MS spectrum 20 V, ESI⁻, HMDB

C

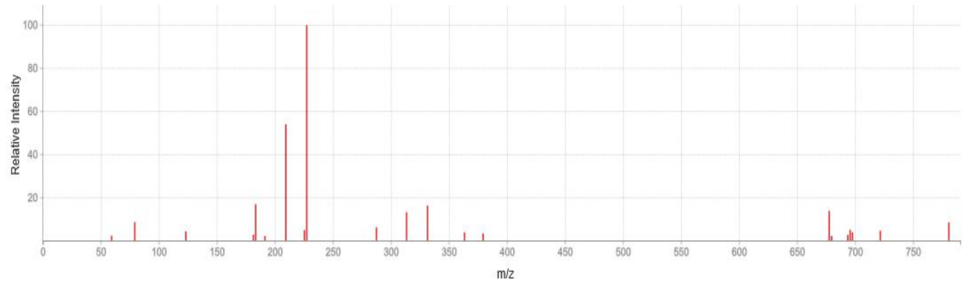

Predicted MS/MS spectrum 40 V, ESI⁻, HMDB

D

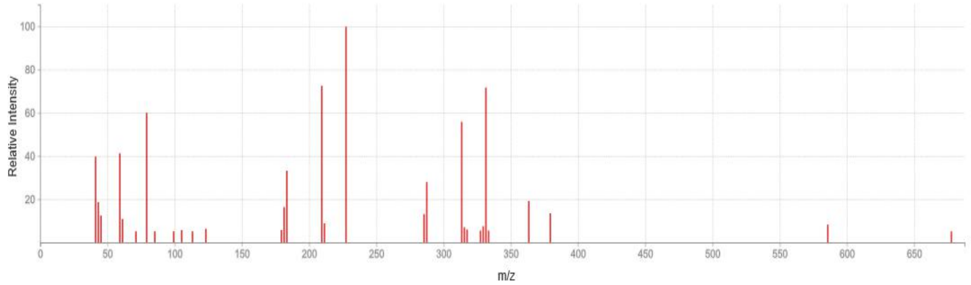

Figure S20. The MS/MS spectra of identified potential marker PC(14:0/22:4(7Z,10Z,13Z,16Z)) (A) and the MS/MS spectrum in HMDB database (B, C, D)

MS/MS Spectrum of identified potential marker PC(14:0/20:2(11Z,14Z)) (t_R: 27.91 min)

A

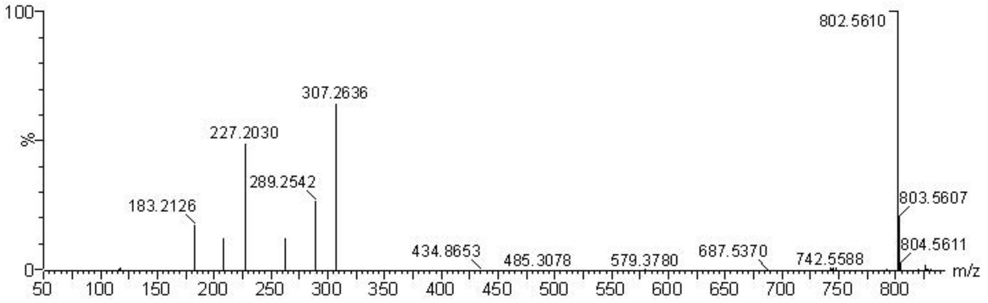

Predicted MS/MS spectrum 10 V, ESI-, HMDB

B

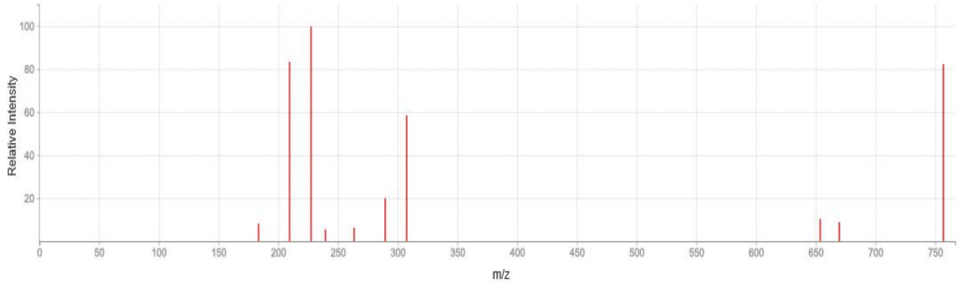

Predicted MS/MS spectrum 20 V, ESI-, HMDB

C

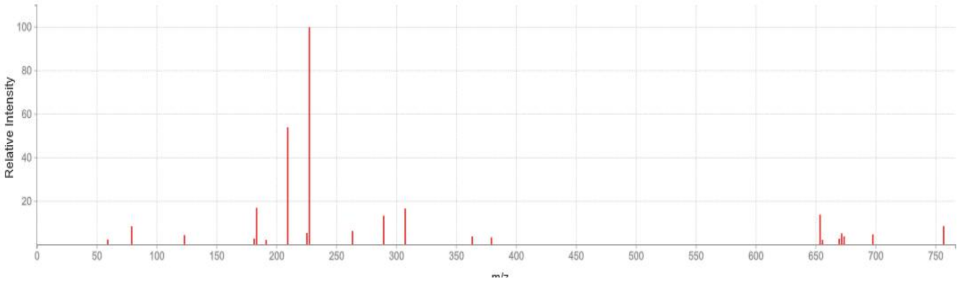

Predicted MS/MS spectrum 40 V, ESI-, HMDB

D

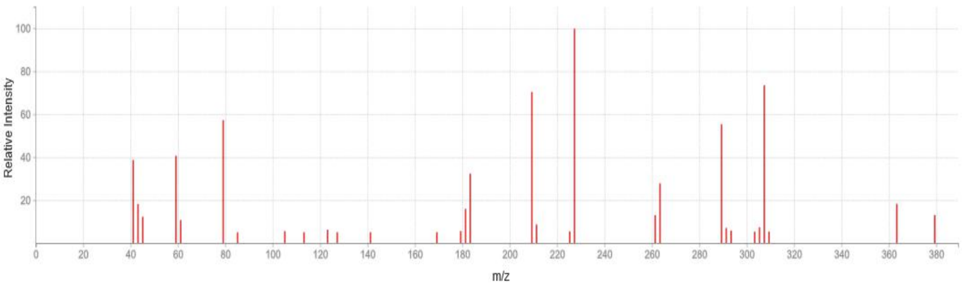

Figure S21. The MS/MS spectra of identified potential marker PC(14:0/20:2(11Z,14Z)) (A) and the MS/MS spectrum in HMDB database (B, C, D)
